# Development and characterization of NILK-2301, a novel CEACAM5xCD3 κλ bispecific antibody for immunotherapy of CEACAM5-expressing cancers

**DOI:** 10.1186/s13045-023-01516-3

**Published:** 2023-12-12

**Authors:** Anja Seckinger, Sara Majocchi, Valéry Moine, Lise Nouveau, Hoang Ngoc, Bruno Daubeuf, Ulla Ravn, Nicolas Pleche, Sebastien Calloud, Lucile Broyer, Laura Cons, Adeline Lesnier, Laurence Chatel, Anne Papaioannou, Susana Salgado-Pires, Sebastian Krämer, Ines Gockel, Florian Lordick, Krzysztof Masternak, Yves Poitevin, Giovanni Magistrelli, Pauline Malinge, Limin Shang, Sonja Kallendrusch, Klaus Strein, Dirk Hose

**Affiliations:** 1 LamKap Bio Alpha AG, Bahnhofstrasse 1, 8808 Pfäffikon, SZ Switzerland; 2 Light Chain Bioscience - Novimmune SA, Chemin du Pré-Fleuri 15, 1228 Plan-les-Ouates, Switzerland; 3 Institute of Anatomy, Leipzig University, Liebigstrasse 13, 04103 Leipzig, Germany; 4 Department of Visceral, Transplantation, Thoracic and Vascular Surgery, University Hospital Leipzig, Liebigstrasse 20, 04103 Leipzig, Germany; 5 Department of Medicine II, University Cancer Center Leipzig (UCCL), Leipzig University Medical Center, Liebigstrasse 22, 04103 Leipzig, Germany; 6 Institute of Clinical Research and System Medicine, Health and Medical University Potsdam, Schiffbauergasse 14, 14467 Potsdam, Germany

**Keywords:** Bispecific antibody, CD3, CEACAM5, Immunotherapy, T-cell engager, Solid cancer

## Abstract

**Background:**

T-cell retargeting to eliminate CEACAM5-expressing cancer cells via CEACAM5xCD3 bispecific antibodies (BsAbs) showed limited clinical activity so far, mostly due to insufficient T-cell activation, dose-limiting toxicities, and formation of anti-drug antibodies (ADA).

**Methods:**

We present here the generation and preclinical development of NILK-2301, a BsAb composed of a common heavy chain and two different light chains, one kappa and one lambda, determining specificity (so-called κλ body format).

**Results:**

NILK-2301 binds CD3ɛ on T-cells with its lambda light chain arm with an affinity of ≈100 nM, and the CEACAM5 A2 domain on tumor cells by its kappa light chain arm with an affinity of ≈5 nM. FcγR-binding is abrogated by the “LALAPA” mutation (Leu234Ala, Leu235Ala, Pro329Ala). NILK-2301 induced T-cell activation, proliferation, cytokine release, and T-cell dependent cellular cytotoxicity of CEACAM5-positive tumor cell lines (5/5 colorectal, 2/2 gastric, 2/2 lung), e.g., SK-CO-1 (*E*_max_ = 89%), MKN-45 (*E*_max_ = 84%), and H2122 (*E*_max_ = 97%), with EC_50_ ranging from 0.02 to 0.14 nM. NILK-2301 binds neither to CEACAM5-negative or primary colon epithelial cells nor to other CEACAM family members. NILK-2301 alone or in combination with checkpoint inhibition showed activity in organotypic tumor tissue slices and colorectal cancer organoid models. In vivo, NILK-2301 at 10 mg/kg significantly delayed tumor progression in colon- and a pancreatic adenocarcinoma model. Single-dose pharmacokinetics (PK) and tolerability in cynomolgus monkeys at 0.5 or 10 mg/kg intravenously or 20 mg subcutaneously showed dose-proportional PK, bioavailability ≈100%, and a projected half-life in humans of 13.1 days. NILK-2301 was well-tolerated. Data were confirmed in human FcRn TG32 mice.

**Conclusions:**

In summary, NILK-2301 combines promising preclinical activity and safety with lower probability of ADA-generation due to its format compared to other molecules and is scheduled to enter clinical testing at the end of 2023.

**Supplementary Information:**

The online version contains supplementary material available at 10.1186/s13045-023-01516-3.

## Background

Immunotherapeutic approaches introduced over the last decades exploiting the patient’s immune system to kill tumor cells have shown benefit in a variety of tumor entities [1–8]. T-cells are a particularly active element of anti-tumor immunity: a T-cell recognizing its antigen presented by a MHC class I molecule at the surface of a tumor cell is activated, starts to proliferate, produces cytokines, and kills the cancer cell [9, 10]. The number of T-cells able to physiologically recognize tumor antigens in the MHC-I context is limited, resulting in restricted intrinsic anti-tumor activity. To circumvent this limitation, two approaches are followed: First, by ex vivo genetically engineered T-cells with a chimeric antigen receptor (CAR), so-called CAR-T-cells, targeting the surface antigen of choice [7, 11–14]. Secondly, by T-cell bispecific antibodies (TCBs), re-directing T-cells to tumor cells independently of the specificity of their T-cell receptor (i.e., MHC-I independent). Both approaches have shown high clinical activity, especially in hemato-oncological diseases like multiple myeloma (e.g., BCMAxCD3) [15–17] or B-cell lymphoma (e.g., CD20xCD3) [18]. TCBs are in principle applicable of the shelf to all patients whose tumor cells express the targeted antigen on their cell surface. As T-cells are re-targeted to all cells expressing this antigen at a high enough level, the antigen targeted by the TCB needs to be as tumor cell specific as possible.

CEACAM5 (CEA, CD66e) was first described in 1965 as specific “carcinoembryonic” antigen of the human gastrointestinal (GI) tract [19, 20]. CEACAM5 belongs to the family of CEA-related cell adhesion molecules (CEACAMs) that comprises 12 closely related proteins in humans [21]. This sequence homology needs to be taken into account when targeting CEACAMs. CEACAM5 is attached to the cell membrane by a glycosyl phosphatidylinositol anchor and released as a soluble form by phospholipase D [22–25]. CEACAMs are involved in a variety of processes, including cell adhesion, intra-/ intercellular signaling, cancer progression, angiogenesis, and metastasis [21, 26, 27]. CEACAM5 is present early in embryonic and fetal development with expression maintained in a limited number of normal adult tissues. Its main site of expression is in columnar epithelial and goblet cells of the colon, particularly in the upper third of the crypt and at the free luminal surface. Together with the presumably lower expression in healthy tissue, this polarized expression pattern is thought to limit on-target off-tumor effects to systemically administered molecules in normal tissue [26, 28–30]. CEACAM5 is (over-) expressed in tumors of epithelial origin, including colorectal, gastric, lung, and pancreatic carcinomas, where it loses its apical expression, resulting in distribution over the entire cell surface [21, 22, 26, 31]. This loss of apical expression makes CEACAM5 accessible to therapeutic antibodies.

Different molecules targeting CEACAM5 for diagnostic or therapeutic purposes have been tested or are currently in clinical trials. These include radiolabeled antibodies, antibody drug conjugates (ADC), and TCBs used both in monotherapy or combination treatment e.g., with checkpoint inhibitors [32–36]. CEACAM5-targeting T-cell engagers conveying the same mechanism of action in clinical trials can be taken as reference for expected class-specific activity and toxicity. These include the MEDI-565 bispecific T-cell engager (BiTE; NCT01284231, NCT02291614) [35, 37] as well as two 2:1 format TCBs: cibisatamab (RG7802, RO6958688; NCT02324257, NCT02650713, and NCT04826003) [34] and RO7172508 (RG6123; NCT03539484) [38]. Signs of activity or formal objective response (the latter for cibisatamab) could be observed, with GI toxicity (on-target) being the main reported adverse event and dose-limiting toxicity in trials for all three compounds. Anti-drug antibodies (ADAs) were found in approximately half of patients in all three trials quoted before, and such ADA-mediated loss of exposure limited clinical efficacy. This may be due to the artificial formats of these CEACAM5-targeting T-cell engagers (i.e., BiTE or especially 2:1 format). Based on these findings, it can be presumed that a bispecific antibody (BsAb) with lower potential of ADA-formation, such as the so-called κλ body format used here [39], could be of significant therapeutic potential. We present here the development and preclinical testing of NILK-2301, a novel CEACAM5xCD3 TCB, for the treatment of CEACAM5-expressing solid tumors.

## Methods

### Affinity to CEACAM5 and CD3

Affinity to CEACAM5 and CD3ε was determined by bio-layer interferometry on an Octet RED96 instrument (Sartorius).

Protein A biosensors (Sartorius) were loaded with NILK-2301 at a concentration of 1 μg/mL for 300 s and a loading signal of 1.2 and 1.3 nm was obtained for all experiments. A 2× serial dilution of CEACAM5 (produced in-house) was prepared in KB buffer (Sartorius), starting at 100 nM, on seven different concentrations, the eighth well of the column corresponding to a blank buffer for reference well subtraction. The association and dissociation steps were monitored for 300 s and 600 s respectively, except for the last experiment with a dissociation recorded for 900 s to better monitor the dissociation. Finally, a regeneration step was applied to reuse biosensors for another interaction.

HIS1K biosensors (Sartorius) were loaded with CD3ε at a concentration of 5 μg/mL for 300 s and a loading signal between 0.6 and 0.7 nm was obtained for all experiments. A 2× serial dilution of NILK-2301 was prepared in KB buffer, starting at 300 nM, on seven concentrations, the eighth well of the column corresponding to a blank buffer for reference well subtraction. The association and dissociation steps were monitored for 300 s and 600 s, respectively. Finally, a regeneration step was applied to reuse biosensors for another interaction.

Data were processed and analyzed using the Data Analysis software (Fortebio). A double reference subtraction was applied and both association, and dissociation steps were fitted using a global 1.1 fitting model.

### Epitope binning

Epitope binning was performed by assessing the binding of the anti-CEACAM5 monoclonal antibody (mAb) to recombinant CEACAM5 protein in the presence of reference antibodies. Six mAbs previously described as binding to different epitopes of human CEACAM5 were produced in house as human IgG1 and/or mouse IgG2a and used as reference antibodies: 1) mAb derived from sm3E (patent US20050147614A1); 2) mAb derived from MEDI-565 (WO2016036678A1); 3) mAb derived from Mab2_VLg5VHg2 (EP3199552A1); 4) mAb derived from CH1A1A-2F1 (US20120251529); 5) mAb derived from variant 1 described in patent WO2017055389; and 6) mAb derived from hMN14 (US 2002/0165360A1).

### Epitope mapping

Human and cynomolgus sequences share 79% of homology, as determined by sequence alignment using Clone Manager software. Based on sequence differences in the domain A2, mutants were designed to identify important residues implicated in the binding of AB73 mAb to human CEACAM5. The mutagenesis was performed in four rounds. Sequences were synthesized by Eurofins and then cloned into pEAK8 vector (Edge Bio). Constructs were transiently transfected in PEAK cells (ATCC), and mutants were expressed at the cell surface. Expression level of all constructs was confirmed using a benchmark antibody (SAR mAb from Sanofi) which binds both human and cynomolgus CEACAM5 proteins. Binding of AB73 anti-CEACAM5 arm was then assessed by flow cytometry.

### Cell lines

CEACAM5-positive colorectal, lung, gastric, or pancreatic cancer cell lines were purchased from ATCC (SK-CO-1, SNU-C1, H508, LS-174T, SW1116, SNU-16, H727, H2122, HPAF-II) or DSMZ (MKN-45) and cultured according to the respective datasheets. CEACAM5-negative A549 cells (ATCC), and two primary epithelial cell lines, i.e., HBEpiC (ScienCell Research Laboratories) and CCD841CoN (ATCC), were used as controls. The number of CEACAM5 antigens/cell was determined by QIFIKIT (Agilent DAKO) according to the manufacturer’s instruction.

### Binding assay

NILK-2301 was tested in a dose range of 0.0128 to 200 nM for its ability to bind to CEACAM5-expressing cell lines using a flow cytometry-based assay. Only the three highest concentrations were tested with CEACAM5-negative cells.

2.5 × 10^5^ cells/well were centrifuged in a 96-well-V-bottom plate and incubated for 20 min at 4 °C, with 100 μL of NILK-2301, anti-CD3 monovalent BsAb or isotype control, in 1/5 serial dilutions covering six concentration points, in phosphate-buffered saline (PBS) containing 2% (w/v) bovine serum albumin (BSA) as binding buffer. Cells were washed twice and 100 μL of secondary Ab (mouse anti-human IgG-Fc-PE, Southern Biotech; 100× diluted) was added and incubated for 20 min.

After washing, cells were resuspended with 150 μL of binding buffer containing Sytox Blue (Thermo Fisher Scientific; 5000× diluted). Cells were analyzed using a CytoFLEX flow cytometer (Beckman Coulter) and raw data extracted by using FlowJo software (BD). Geometric mean fluorescence intensity (MFI) was extracted from the “live cell” gate and plotted against the concentrations of NILK-2301 and control antibody using Prism software (GraphPad).

Besides, directly labeled candidates were used to assess the binding to corpuscular blood components in whole blood samples from healthy donors (Centre de transfusion sanguine Genevois, Geneva, Switzerland). Data acquisition and analysis were performed as described above.

Binding to other CEACAM family members was assessed using transfected PEAK cells. In brief, the full-length DNA sequence coding for human CEACAM proteins was cloned into pEAK8 vector (Edge Bio) and transfected into PEAK cells (ATCC). Transiently expressing cells were used 48 h-72 h post-transfection, following protein expression level determination by flow cytometry using commercially available CEACAM-specific antibodies (Additional file 1: Table S1).

### Receptor occupancy (RO)

NILK-2301 was tested at a dose range of 0.03 to 6000 nM (LS-174 T and SNU-C1), and from 0.003 to 600 nM with SK-CO-1, for its ability to bind and saturate CEACAM5 expressed on these cell lines.

Binding was analyzed by using flow cytometry as described above. RO was calculated for each NILK-2301 concentration using the following formula:

$$RO\% = \left( {MFI \, at \, a \, tested \, NILK - 2301 \, concentration/max. \, MFI} \right) \times 100\%$$

The maximum MFI is the highest MFI obtained within the range of NILK-2301 concentrations tested for the respective cell lines.

### T-cell dependent cytotoxicity (TDCC) assay based on LDH release

PBMCs from healthy donors were used as a source of effector T-cells. At least three donors were used for each cell line tested.

PBMCs was centrifuged at 300*g* for 5 min and the cell pellet resuspended in Roswell Park Memorial Institute (RPMI) 1640 medium supplemented with 2% fetal calf serum (FCS), 2 mM L-glutamine, 25 µg/mL gentamicin at a density of 2 × 10^6^ cells/mL. 1× 10^5^ PBMCs were plated in ultra-low attachment sterile 96-well round bottom plates (Corning) and 1× 10^4^ target cells added.

Antibodies were diluted in RPMI-1640 containing 2% (v/v) FCS at 4× final concentrations and 25 µL of the diluted antibody was added to the plate. The highest final concentration was 100 nM, followed by seven 1/5 serial dilutions. Wells containing only effector cells mixed with target cells, or target cells only, served as controls. Plates were incubated at 37 °C for 48 h.

For readout, 5 µL of lysis buffer (Cytotoxicity detection kit PLUS (LDH), Roche) was added to wells containing only the targets cells and incubated for at least 5 min. The lysis step was verified by visual inspection under the microscope. These wells served as positive control of 100% target cell lysis.

For MKN-45 cells, 50 μL of TDCC sample from each well was transferred to a clear flat-bottom plate and mixed with 50 μL of PBS by 3–4 times of gentle up and down pipetting to adapt for a higher LDH content compared to other cell lines. For the other cell lines, 100 μL of the TDCC sample was transferred directly without dilution.

In the meantime, the detection mixture was prepared according to the manufacturer’s instructions. 100 μL of the freshly prepared detection mixture was added to the plate containing the diluted TDCC samples and incubated at room temperature (RT), protected from light, for 15 min. After incubation, 50 μL of stop reagent was added. Optical density was measured using a microplate reader at 490 nm (Spectra i3Max). The percentage of specific lysis was calculated by using the following equation:

$$Specific \, lysis\% = \,sample \, value - \left( {effector + target} \right)/\left( {max. \, target - target} \right)*100\%$$

Effector + target = baseline without Ab; max. target = target cells with lysis buffer; target = target cells only (spontaneous LDH release). Due to variability in spontaneous LDH release, samples that do not induce TDCC may have slightly lower LDH release than effector + target, and thus result in a negative specific lysis% using the above formula.

Specific lysis results were then reported in GraphPad Prism to establish potency curves.

### T-cell activation and proliferation

T-cell activation was analyzed by assessing the expression of CD69 (early activation marker) and CD25 (late activation marker) on CD4^+^ and CD8^+^ T-lymphocytes. Cells from TDCC assay samples after 48 h of incubation were collected; duplicates were pooled to obtain sufficient cell numbers.

After centrifugation, cell pellets were resuspended in 30 μL Fc block (BD Biosciences) diluted at 1/20 in PBS containing 2% BSA (w/v) (binding buffer) to avoid non-specific binding to FcγR-expressing cells. Plates were then incubated at RT for 10 min. The following antibodies were used: anti-hCD45-V500 (BD Biosciences), anti-hCD69-FITC, anti-hCD8-PerCP-Cy5.5, anti-hCD25-PE (all from Biolegend), and anti-hCD4-APC (eBioscience). Antibodies were diluted in cold binding buffer diluted 1/2 with Brilliant buffer (BD Biosciences), then added into each well (100 µL) and mixed with cells. Plates were incubated for 20 min at 4 °C.

After washing, cells were resuspended in 100 μL containing eFluor780 diluted at 1/1000 in cold PBS to exclude dead cells from analysis. After 30 min of incubation at 4 °C, cell pellets were washed twice and resuspended in 150 μL of binding buffer for fluorescence analysis using the CytoFLEX flow cytometer (Beckman Coulter). Data were extracted and analyzed by using FlowJo software.

To assess T-cell proliferation, PBMCs were stained with 5 µM CellTrace violet (Thermo Fisher Scientific) for 20 min at 37 °C. After incubation, PBMCs were washed twice and used in the TDCC assay as described above, with incubation for six instead of two days. Cells from duplicates were pooled to obtain enough events for flow cytometric analysis. Cell staining and data analysis were performed as described above.

### Cytokine quantification from TDCC assays

A U-PLEX Immuno-Oncology Group1 kit from Meso Scale Discovery (MSD) was used to quantify granzyme B, interferon (IFN) γ, interleukin (IL-) 1β, IL-2, IL-6, IL-8, IL-10, IL-12p70, tumor necrosis factor (TNF) α, and granulocyte–macrophage colony-stimulating factor (GM-CSF) in supernatants from TDCC assays according to the manufacturer’s instruction. Supernatants and standard point samples were loaded to 10-spot 96-well U-plex plates coated with U-plex linker coupled antibodies and incubated for one hour at RT. Afterward, corresponding detection antibodies diluted at 1/600 in diluent 3 from the kit were added and incubated for one hour at RT. Before acquisition, plates were filled with “read buffer” diluted 1/2 in deionized water. Results were acquired using the MSD-Discovery Workbench 4.0 embedded software and data plotted with GraphPad Prism software.

### TDCC assay based on ATP quantification

1 × 10^4^ target cells were seeded in flat-bottom 96-well plates for 24 h at 37 °C. On the day of the assay, PBMCs were centrifuged, and the cell pellet was resuspended in RPMI-1640 medium containing 10% heat-inactivated FCS, 2 mM L-glutamine, 1 mM sodium pyruvate, 10 mM HEPES, 1× non-essential amino acid (all from Sigma-Aldrich), 50 µM 2-mercapto ethanol (Gibco) and 25 µg/mL gentamicin (Sigma-Aldrich). 1 × 10^5^ PBMCs were then added to the target cells seeded the day before. Tested antibodies were diluted at 4× final concentrations, and 50 µL added to the wells. The highest final concentration was 100 nM, followed by nine 1/5 serial dilutions. Wells containing only effector cells mixed with target cells served as controls to calculate the specific killing. Plates were incubated at 37 °C for 72 h.

For the readout, plates were shaken on a rocking platform to resuspend the PBMCs in the supernatant. Subsequently, plates were washed twice with PBS to remove all PBMCs. In the meantime, the reagent from the kit (CellTiter-Glo® viability assay, Promega) was reconstituted, before adding 100 µL to each well. Incubation was performed for 10 min at RT.

The luminescence signal coming from the adenosine triphosphate (ATP) present in the remaining live target cells was measured using a Spectra i3Max microplate reader. The percentage of specific lysis was calculated using the following equation:

$$Specific \, lysis\% = 1 - sample \, value/\left( {effector + target} \right) \times 100\%$$

Effector + target = baseline without Ab.

Specific lysis results were analyzed using GraphPad Prism software.

### Whole blood cytokine release

10 μL of antibodies diluted (20×) in PBS (30 and 300 nM final concentrations) were added to U-bottom 96-well plates, in triplicate. Subsequently, 190 μL of freshly collected whole blood (undiluted) were added to the diluted antibodies (without mixing to avoid mechanical activation of cells). After 24 h of incubation at 37 °C, plates were centrifuged and supernatants (80 μL) were collected, transferred to another plate and frozen until testing. IFNγ, IL-6, and TNFα were measured as previously described [40, 41]. Data were plotted with GraphPad Prism software.

### PBMC cytokine release and T-cell activation

PBMCs were isolated from whole blood of healthy donors and incubated overnight at 37 °C, 5% CO_2_. The next day, 40 μL of antibodies (5×) diluted in medium (3, 30, and 300 nM, final concentrations) were put in ultralow attachment flat-bottom 96-well plates, in duplicates. 2 × 10^5^ PBMCs (160 μL) were added in each well followed by centrifugation and incubation at 37 °C. After 48 h, plates were centrifuged and supernatants were collected, transferred to another plate and frozen until testing. IFNγ, IL-2, IL-10, and TNFα were measured as previously described [31, 32]. Data were plotted with GraphPad Prism software.

After collection of the supernatants, plates were centrifuged to pellet the cells and the latter transferred to a V-bottom 96-well plate (duplicates were pooled). 25 μL/well of Fc Block (1/20 in FACS buffer) were added to each well and the plates were incubated for 10 min at RT. 25 μL/well of antibody cocktail (anti-CD3-APC, anti-CD69-FITC, anti-CD25-PC5, 3 μL each/well) were added. Plates were incubated for 15 min at RT and 150 μL/well of FACS buffer were added. After centrifugation, supernatants were discarded, and cells resuspended in 200 μL/well of CellFix (1/10 in H_2_O). Plates were acquired on CytoFLEX flow cytometer, data were exported from CytExpert software and plotted using GraphPad Prism.

### Organoid culture and TDCC assay

Experiments were performed in collaboration with HUB ORGANOIDS, Utrecht, the Netherlands. Colorectal cancer organoids were seeded in 70% matrigel (Corning) and expanded on medium containing Ad-DF+++ (DMEM/F12 (Thermo Fisher) supplemented with 2 mM GlutaMax (Thermo Fisher), 10 mM HEPES (Thermo Fisher), penicillin/streptomycin (Thermo Fisher), 1.25 mM N-Ac (Sigma-Aldrich), 500 nM A83-01 (Tocris), 1× B27 supplement (Thermo Fisher), 2% Noggin (UPE), 5 mM nicotinamide (Sigma-Aldrich), 50 ng/mL EGF (Prepotech), 5 nM Gastrin (Bio-Techne), 50 μg/mL Primocin (Invivogen), 250 ng/mL Rspondin-3 (Tocris), and 500 nM SB202190 (Sigma-Aldrich). One model required medium, which contained 50% Wnt3a conditioned-medium produced in-house. Organoids were passaged every 6–7 days, and their medium refreshed every 2–3 days.

Flow cytometry was used to confirm cell surface expression of CEACAM5 on selected organoids; MKN-45 cells (DSMZ) were used as positive control. After dissociation using Accutase (Life Technologies), single cells were washed with FACS buffer and stained with anti-CEACAM5 (Santa Cruz) for 20 min at 4 °C. Control anti-CD19 (R&D systems) was used to discriminate between positive and background signals. Cells were washed with FACS buffer and recorded with a MACSQuant analyzer 10 flow cytometer (Milteny Biotec). Data were analyzed by MACSQuantify10 or FlowLogic software provided by Miltenyi Biotec. Live cells were selected from the singlet population by Sytox Blue (Thermo Fisher).

To quantify the number of antigens present at the cell surface, QIFIKIT assay (Agilent DAKO) was used according to manufacturer’s instructions.

PBMCs were isolated from whole blood by diluting it 1:1 with PBS/EDTA and separating it through a density gradient obtained with Lymphoprep (StemCell Technologies). The PBMC pellet was resuspended in cold recovery cell freezing medium (Thermo Fisher) at a density of 1 × 10^7^ cells/mL and aliquoted in cryovials (30 × 10^6^/cryovial).

Organoids were split in a 1:1 ratio on the day prior to the TDCC assay. On the day of the assay, organoids were harvested and at least 2 × 10^4^ organoids were digested to single cells by Accutase before counting. After centrifugation, the resulting organoid pellet was resuspended in NucBlue diluted 1:10 in colon medium, followed by a 20 min incubation at 37 °C. Subsequently, 1 mL of Ad-DF+++ was added and organoids were carefully resuspended. Next, organoids were washed with colon medium supplemented with 5 μM ROCK inhibitor. After centrifugation, organoid pellets were resuspended in co-culture medium (45% colon medium, 45% ImmunoCult-XF T Cell Expansion Medium (StemCell Technologies), 10% matrigel supplemented with 10 μM ROCK inhibitor, 150 IU/mL IL-2 and 10,000× diluted IncuCyte Caspase 3/7 reagent [Essen Bioscience]). After counting, the organoid suspension was seeded in 100 μL per well of 96-well ULA plate.

Allogeneic PBMCs were thawed prior to the experiment. After counting, PBMCs were seeded on top of the organoids in 100 μL at an effector:target ratio of 8:1. NILK-2301 was tested at 0.3, 1 and 5 μg/mL. Staurosporine at 10 µM was used as positive control, a monovalent anti-CD3 antibody served as negative control.

Co-cultures were imaged at regular intervals for up to 62 h using the Operetta High Content Screening System (PerkinElmer). Harmony 4.9 software (PerkinElmer) was used for data analysis. An analysis pipeline was generated allowing the identification of organoids (based on NucBlue signal) with a minimum diameter of 40 μm. Per identified object, the mean intensity of the caspase 3/7 fluorescent dye was quantified. To correct for background signals, the mean caspase 3/7 intensity in a region of 10 pixels around the objects was subtracted from the mean caspase 3/7 intensity for each organoid. The average of two wells was plotted as a mean with standard deviation.

IFN-γ production was determined in culture supernatants by using ELISA (R&D systems) according to the manufacturer’s instructions. Absorbance was measured using a Spark 10 M (Tecan) plate reader.

### Patient-derived tissue culture (PDTC) model

NILK-2301 monotherapy (15 µg/mL) and different combinations with immune checkpoint inhibitors, i.e., pembrolizumab (Merck/MSD; 3 µg/mL), nivolumab (Bristol-Myers Squibb; 3 µg/mL), and ipilimumab (Bristol-Myers Squibb; 1 µg/mL), were assessed using tumor specimens from patients diagnosed with colon (*n* = 21), gastric (*n* = 16), or lung cancer (*n* = 17). The study was approved by the ethics committee of the Medical Faculty, University of Leipzig (# 317/13-ek). All patients had given prior written informed consent.

PDTCs were prepared according to standardized protocols followed by compound exposure as published [42]. In brief, tissue slices were cultured 24 h in RPMI-1640 (Gibco), containing 10% fetal bovine serum (Gibco), 1% amphotericin B (Carl Roth), 1% penicillin/streptomycin (Gibco), and 1% L-glutamine (Gibco). Media was changed after 6 h and 24 h. After this pre-cultivation phase, PDTCs were incubated with NILK-2301 and combinations as indicated for 72 h. A semi-automatized readout using fluorescent stain-specific segmentation algorithms for ImageJ was performed to quantify changes in tumor cell viability, proliferation (Ki67), and apoptosis (cleavedPARP). The staining of CD3 and CD68 was performed only in some experiments and was not quantitatively evaluated. The following antibodies were used: AE1/3 (BioGenex; mouse, 1:100), cPARP (Abcam; rabbit, 1:100), Ki67 (DCS; rabbit, 1:200), CEACAM5 (RnD Systems; mouse, 1:100), CD3 (Bio-Rad Laboratories; rat, 1:200), and CD68 (DAKO; mouse, 1:250). Tissue was stained with diaminobenzidine (Sigma-Aldrich) and counterstained with hematoxylin. The following secondary antibodies were used: biotinylated goat anti-rat (Vector laboratories, 1:100) and biotinylated goat anti-mouse (Sigma-Aldrich, 1:100) diluted in 0.3% PBS/TritonX.

Five pictures per slice (3 × 5 pictures/condition) were taken manually by using an Olympus BX51 microscope.

Only pictures with > 2% tumor cells (Hoechst 3342-, AE1/3-positive) were considered for further quantification of apoptotic and proliferating tumor cell fractions. Due to experimental variance that was similarly described in Sönnichsen et al. [42], each condition was normalized to the control condition (CTR = 1). Effects on tumor fraction (TF), tumor apoptosis (TA), and proliferation (TP) were combined into one final score. The following formula was used to calculate the overall outcome:

$$1 + TF - \left( {1.2*TA} \right) + \left( {0.8*TP} \right).$$

For statistical analysis, mean values for conditions were calculated using mean slice values and standard error of the mean (SEM). For combination of different experiments, mean values and SEM were calculated from mean condition values. GraphPad Prism 8 (GraphPad software) was used for calculating one-way analysis of variance with Benjamin-Hochberg post-test correction. *P* < 0.05 was considered to be significant.

### Xenograft models

Animal studies were performed in accordance with the Swiss Veterinary Office guidelines and approved by the Cantonal Veterinary Office (Geneva, Switzerland). Study endpoints included tumor volume > 1500 mm^3^, body weight loss > 15%, and other graft-versus-host-disease symptoms.

In the HPAF-II model, 7–8-week-old NOG female mice (NOD/Shi-scid/IL-2Rg^null^, Taconic Biosciences) were injected subcutaneously (SC) with 1 × 10^6^ HPAF-II target cells, followed by intravenous (IV) injection of 1 × 10^7^ PBMCs on day 7. Treatment with NILK-2301 IV at 10 mg/kg weekly or vehicle control (PBS; *n* = 8 each) was started on day 11, when the average tumor volume of mice reached 150 mm^3^.

NILK-2301 was tested in a second in vivo model, where 1 × 10^6^ LS-174T target cells and 1 × 10^6^ PBMCs were co-grafted SC in female NOG mice of 7–8 weeks of age. Treatment was started at day 7 post co-grafting; NILK-2301 was dosed IV at 10 mg/kg twice/week, and PBS served as vehicle control (*n* = 8 each).

Mice were controlled daily for clinical symptoms and potential adverse events. Tumor measurement was performed by caliper twice/week.

### Pharmacokinetic (PK) and tolerability studies

#### Cynomolgus monkeys

A single-dose study with 6-week observational period was conducted in cynomolgus monkeys (*Macaca fascicularis*) to evaluate in vivo tolerability and pharmacokinetic profile of NILK-2301 when injected IV or SC (Accelera S.r.l., Italy). NILK-2301 was produced and purified as described in Fischer et al. [39] and as summarized in Additional file 1: Figure S1. The purified bispecific antibody was formulated at either 0.5 or 20.9 mg/mL in 25 mM Histidine, 125 mM NaCl, pH 6.0 supplemented with 0.02% Polysorbate 80. Both batches present 98.6% of IgG monomer (1.4% of aggregates and 0% of fragments) and tested negative for bacterial endotoxins (LAL test). Animals in groups 1 and 2 (two males and two females per group) received a single IV dose of NILK-2301 at 0.5 or 10 mg/kg, respectively, and were observed for six weeks regarding changes in clinical signs (including local tolerance), body weights, food intake, safety pharmacology, clinical pathology (hematology, coagulation, and clinical chemistry), immunophenotyping, and PK. At the end of this period (test period 1), animals in group 1 were sacrificed. Necropsies including recording of terminal body weights, macroscopic examination, and tissue collection were conducted at the end of the test period. No microscopic examinations were conducted since no lesions were observed upon macroscopic examinations and no unscheduled deaths occurred during the study. Animals in group 2 were reused and allocated to group 3, for a 6-week evaluation of PK upon SC dosing at 20 mg/kg (test period 2), after which they were also sacrificed.

For the two IV groups, PK samples were collected at pre-dose, and 0.25, 1, 4, 8, 24, 48, 72, 120, 168, 336, 504, 672, 840, and 1008 h post-dose. For the SC group, PK samples were collected at pre-dose, 0.5, 1, 4, 8, 24, 48, 72, 120, 168, 336, 504, 672, 840, and 1008 h post-dose.

#### Human FcRn Tg32

B6.Cg-Fcgrt^tm1Dcr^Tg(FCGRT)32Dcr/DcrJ mice (The Jackson Laboratory) of 7–8 weeks of age were injected with a dose of 0.5 (*n* = 11) or 10 mg/kg (*n* = 12) NILK-2301 IV once in the tail vein. Mice were controlled daily for clinical symptoms and potential adverse events. Furthermore, mice were weighed at timepoint 0, 24, 48, 96, 168, 240, 336, 504, and 672 h. Percentage of body weight was calculated compared to body weight at *T* = 0 (100%).

For PK analysis, blood was collected three times in each mouse to cover the following timepoints: 0.25, 1, 4, 8, 24, 48, 72, 120, 168, 336, 504, and 672 h post-dose.

### PK analysis

Quantification of NILK-2301 in serum samples was performed at Light Chain Bioscience (Switzerland) using a generic pharmacokinetic assay based on MSD. Raw data and corresponding concentrations were generated with MSD instrument using MSD WorkBench 4.0 software for acquisition and analysis.

Non-compartmental analysis was performed at Calvagone Sarl (France) using SAS software on data from all animals with available concentrations.

## Results

### Development of NILK-2301

The κλ body platform [39] was used to generate a human CEACAM5xCD3 TCB selectively targeting CEACAM5 on tumor cells and CD3 on T-cells for the treatment of CEACAM5-positive solid cancers. Firstly, a panel of anti-CD3 antibody arms was generated, all sharing the same common heavy chain and being derived from the mouse anti-human/-cyno CD3ε antibody SP34 [43]. This antibody was humanized and diversified to obtain novel unique sequences of different affinity. One variant, termed L3-1, was found to have similar affinity for CD3 as the parental antibody and was selected to be used as anti-CD3 arm. To generate compatible anti-CEACAM5 arms, phage display libraries were built based on the L3-1 VH, with high diversity in the light chain variable sequences. These libraries were selected and screened to identify suitable anti-CEACAM5 antibody arms, which were subsequently combined with the L3-1 anti-CD3 arm. Binding to Fc-gamma receptors (FcγRs) was abrogated by the “LALAPA” mutations (Leu234Ala, Leu235Ala, Pro329Ala [44–46]). The introduction of this triple mutation intentionally abrogates binding to FcγRs but maintains binding to the human/cynomolgus neonatal Fc receptor (FcRn), ensuring IgG-like half-life in circulation. κλ bodies were then generated by transfecting a plasmid encoding for the three antibody chains into a suitable host cell line followed by a three-step affinity purification process resulting in the isolation of highly pure bispecific antibodies (Additional file 1: Figure S1) as described in [39]. Generated κλ bodies were tested in several in vitro assays, to assess their activity and safety, and the most promising candidates were subjected to an affinity maturation process (lead optimization). Following the in-depth characterization of lead-optimized candidates, including testing in mouse models and preliminary manufacturability assessment, a candidate matching the target product profile was identified, selected as clinical lead candidate, and termed NILK-2301.

### Characterization of NILK-2301

#### Affinity for CEACAM5, CD3, and Fc receptors

The CEACAM5-targeting arm of NILK-2301, named AB73, binds human CEACAM5 with 5 ± 2 nM affinity. NILK-2301 does not show cross-reactivity with cynomolgus CEACAM5. The CD3-targeting arm binds human CD3 with a measured affinity ranging from 28 ± 3 nM to 95 ± 8 nM, depending on the recombinant protein used. NILK-2301 binds to cynomolgus CD3 with less than 10% difference as compared to human CD3 in the same assay. The higher binding affinity to CEACAM5 as compared to CD3 conveys the intended safety feature of limiting unspecific T-cell activation by CD3-stimulation. NILK-2301 binds to FcRn with an affinity of 3.7 ± 0.6 to 9 nM. Binding affinity to other human and cynomolgus receptors was assessed for the parental antibody of NILK-2301, named AB17L3-1/N, which contains the identical Fc-part (LALAPA-mutated human IgG1 Fc).

#### Epitope binning and epitope mapping of the CEACAM5-binding site

To determine the CEACAM5-binding region and key residues, epitope binning was performed by assessing the binding of the anti-CEACAM5 mAb to recombinant CEACAM5 protein in the presence of several reference antibodies. NILK-2301 was found to compete with a reference antibody, which is known to bind to the A2 domain of CEACAM5. To determine the binding epitope of NILK-2301, two mutagenesis strategies were applied, identifying two residues were as essential for AB73-binding to human CEACAM5: D402 and I408.

#### Absence of NILK-2301 binding to other CEACAMs

The absence of cross-reactivity of NILK-2301 toward other CEACAM proteins was confirmed by flow cytometric binding assay using PEAK cells transfected with different members of the CEACAM family. The monovalent anti-CD3 BsAb was used as isotype control, while non-transfected PEAK cells were used as negative binding control (Additional file 1: Figure S2 and Additional file 1: Table S1).

### In vitro activity and mechanism of action

#### Binding to CEACAM5-positive and -negative cell lines

NILK-2301 binds to all CEACAM5-positive cell lines (Additional file 1: Figure S3). No unspecific binding was observed to the CEACAM5-negative A549 cell line or to cells isolated from normal colon tissue (CCD841CoN) and human bronchi (HBEpiC), respectively. The CEACAM5 density on the CEACAM5-positive tumor cells ranged from 257,000 CEACAM5/cell on SK-CO-1 to 6,5000 CEACAM5/cell on SW1116 (Additional file 1: Table S2).

The monovalent BsAb variant carrying the same CD3 arm as NILK-2301 but paired with a non-binding arm showed no binding to CEACAM5-positive cell lines, confirming that NILK-2301 bound to CEACAM5-positive cell lines through its CEACAM5-binding arm.

Directly labeled NILK-2301 was used to assess the binding to corpuscular blood components without the need of using a secondary detection antibody, which could cause some unwanted background. Overall, no binding was observed on the tested cell populations, except for T-cells, which do express CD3 and for which the binding was expected (data not shown).

#### NILK-2301-mediated TDCC of CEACAM5-positive tumor cells

To assess NILK-2301 activity and mechanism of action, we first addressed killing of CEACAM5-positive tumor cell lines co-incubated with PBMCs from healthy human donors (containing approximately 50% of CD3^+^ T-cells) as effector cells. Two timepoints, i.e., 48 h and 72 h were analyzed.

**Fig. 1:**
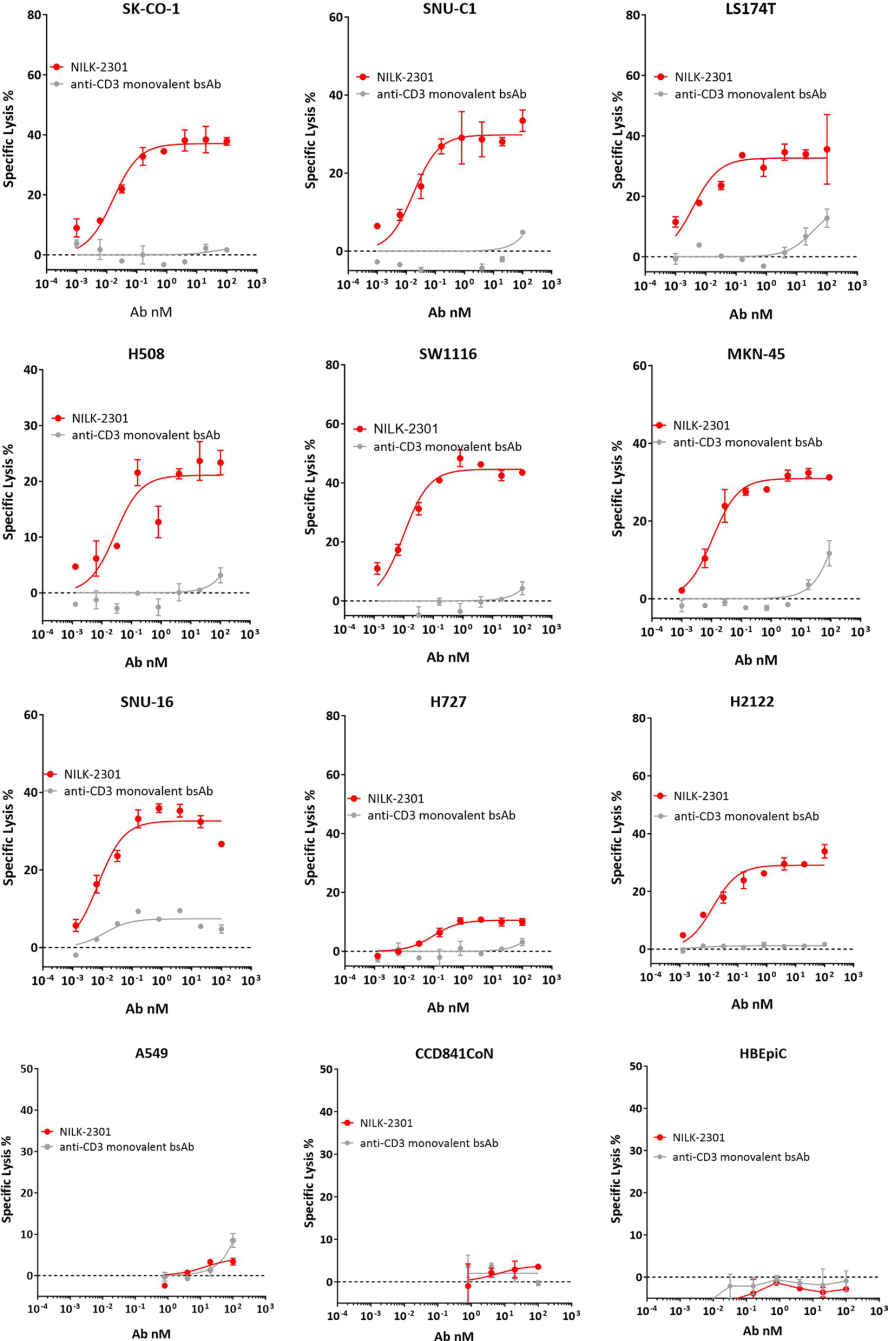
NILK-2301 mediated killing of CEACAM5-positive tumor cell lines based on LDH release after 48 h of incubation. Data for SK-CO-1, SNU-C1, LS-174 T, H508, SW1116, MKN-45, SNU-16, H727, and H2122 are shown. No killing was observed for CEACAM5-negative cell line A549 and CCD841CoN cells, isolated from normal colon, as well as HBEpiC, isolated from normal bronchial tissue. Please refer to Additional file 1: Figure S4 for TDCC data based on ATP quantification after 72 h of incubation

After 48 h, NILK-2301 induced dose-dependent killing of all CEACAM5-positive cell lines regardless of tumor type (Fig. 1) as determined by LDH release, with half maximum effective concentrations (EC_50_) ranging from 0.008 nM (H2122) to 0.15 nM (H508) (Table 1). The CD3 monovalent BsAb variant, which does not carry the CEACAM5-binding arm, did not induce relevant killing of the tested tumor cells (killing < 4%, eightfold less compared to NILK-2301, LS-174T), confirming that NILK-2301-mediated killing is CEACAM5-dependent. No correlation between EC_50_ and CEACAM5 density on-target tumor cells was observed (*R*^2^ = 0.001). NILK-2301 did not induce killing of a CEACAM5-negative cell line (A549) or cells isolated from normal colon (CCD841CoN) or bronchial tissue (HBEpiC), respectively (Fig. 1).

**Table 1:** EC_50_ and maximal plateau from TDCC assays [%] with CEACAM5-positive tumor cell lines

	NILK-2301	
	EC_50_ nM	Max plateau
SK-CO-1	0.06 ± 0.07	43.52 ± 7.26
SNU-C1	0.02 ± 0.01	24.99 ± 12.47
H508	0.15 ± 0.17	14.1 ± 7.56
SW1116	0.033 ± 0.03	38.03 ± 6.71
LS-174 T	0.059 ± 0.09	26.67 ± 9.09
MKN-45	0.02 ± 0.03	27.87 ± 6.31
SNU-16	0.08 ± 0.11	20.92 ± 13.59
H2122	0.008 ± 0.005	25.5 ± 3.7

Readout using LDH release after incubation for 48 h

To assess the impact of longer incubation on maximum specific lysis, a TDCC assay was performed using ATP quantification as measure for the number of remaining living tumor cells. Three tumor cell lines, i.e., SK-CO-1, MKN-45 and H2122, were used.

As expected, a longer incubation with NILK-2301 resulted in a higher specific lysis, with the maximum plateau of specific lysis reaching more than 80% for all three cell lines tested, i.e., 89.1% (SK-CO-1), 84.7% (MKN-45), and 97.2% (H2122) (Additional file 1: Figure S4).

#### NILK-2301-induced T-cell activation

**Fig. 2:**
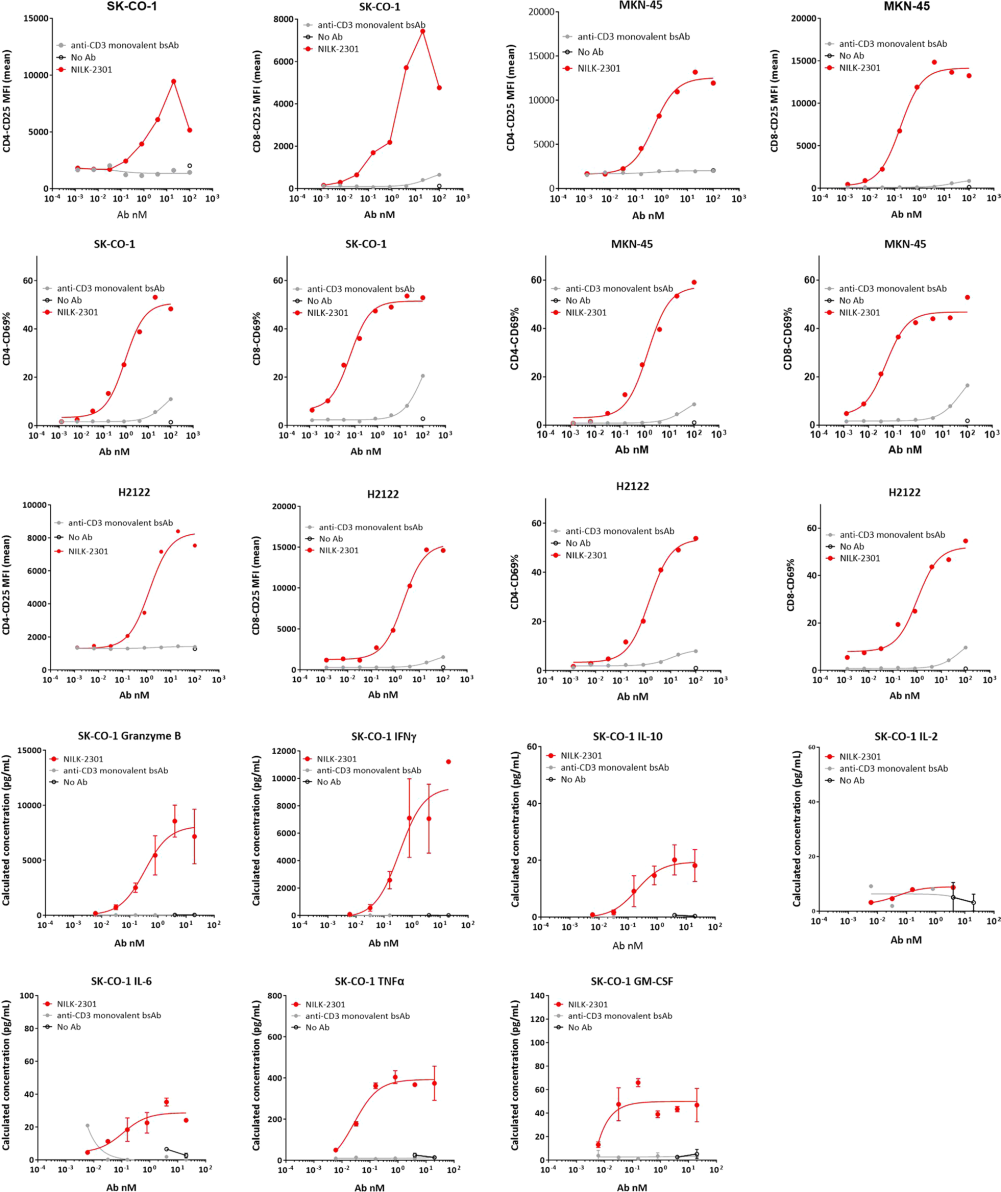
T-cell activation marker analysis for CD69 (late activation marker) and CD25 (early activation marker) as well as cytokine release. T-cell activation after 48 h of incubation in the TDCC assay was assessed using SK-CO-1, MKN-45, and H2122. Cytokine and granzyme B release in supernatants from TDCC is exemplarily shown for SK-CO-1. BsAb, bispecific antibody

To assess the mechanism of action, we first investigated the T-cell activation markers CD69 (early) and CD25 (late) were investigated by flow cytometry in the TDCC assay after 48 h of incubation in the TDCC assay with flow cytometry. With all cell lines tested, NILK-2301 induced dose-dependent activation of both CD4^+^ and CD8^+^ T-cell subpopulations, as depicted by the increased expression of CD25/CD69 at the T-cell surface (Fig. 2).

Secondly, we quantified release of cytokines (i.e., IFNγ, IL-10, IL-2, IL-6, TNFα, GM-CSF, IL-12p70, IL-1b and IL-8) and granzyme B by using the MSD platform in supernatants collected from the TDCC assay with the tumor cell lines SK-CO-1, MKN-45 and H2122 after 48 h. With the exception of IL-2, increased levels were observed in a dose-dependent manner when NILK-2301 was added compared with the monovalent anti-CD3-BsAb control (Fig. 2).

#### NILK-2301-induced T-cell proliferation

**Fig. 3:**
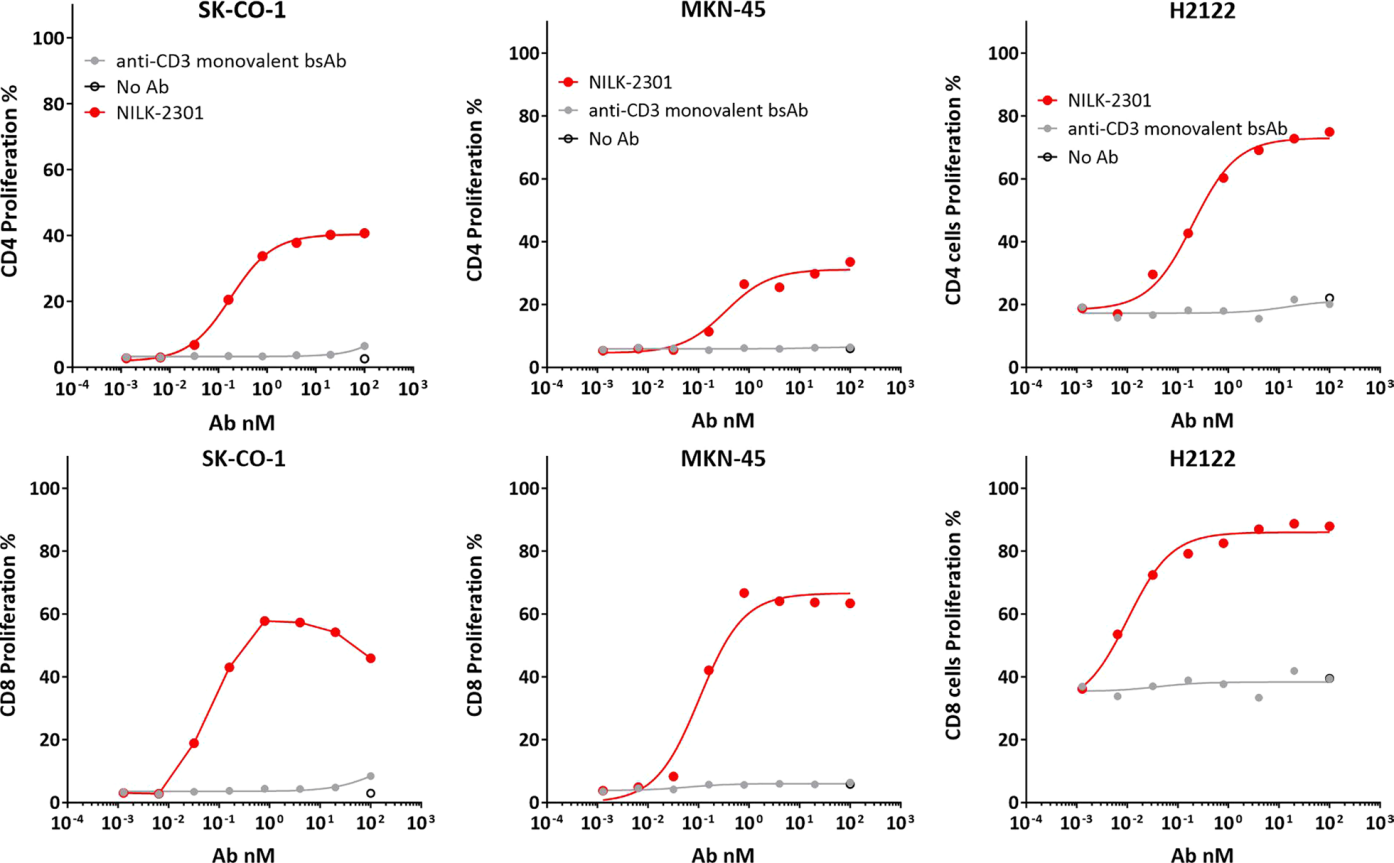
Induction of T-cell proliferation. CD4 (upper panel) and CD8 (lower panel) T-cell proliferation analysis after six days of co-culture with CEACAM5-positive cell lines SK-CO-1, MKN-45, and H2122. BsAb, bispecific antibody

Because NILK-2301 induced T-cell activation and cytokine release, T-cells were found to proliferate significantly better than the anti-CD3 monovalent BsAb control during TDCC assays after six days of incubation for all three tumor cell lines investigated, i.e., SK-CO-1, MKN-45, and H2122 (Fig. 3).

#### Whole blood and PBMC cytokine release and T-cell activation

To assess tumor cell-independent T-cell activation, we exposed whole blood and PBMCs from healthy donors to different concentrations of NILK-2301.

**Fig. 4:**
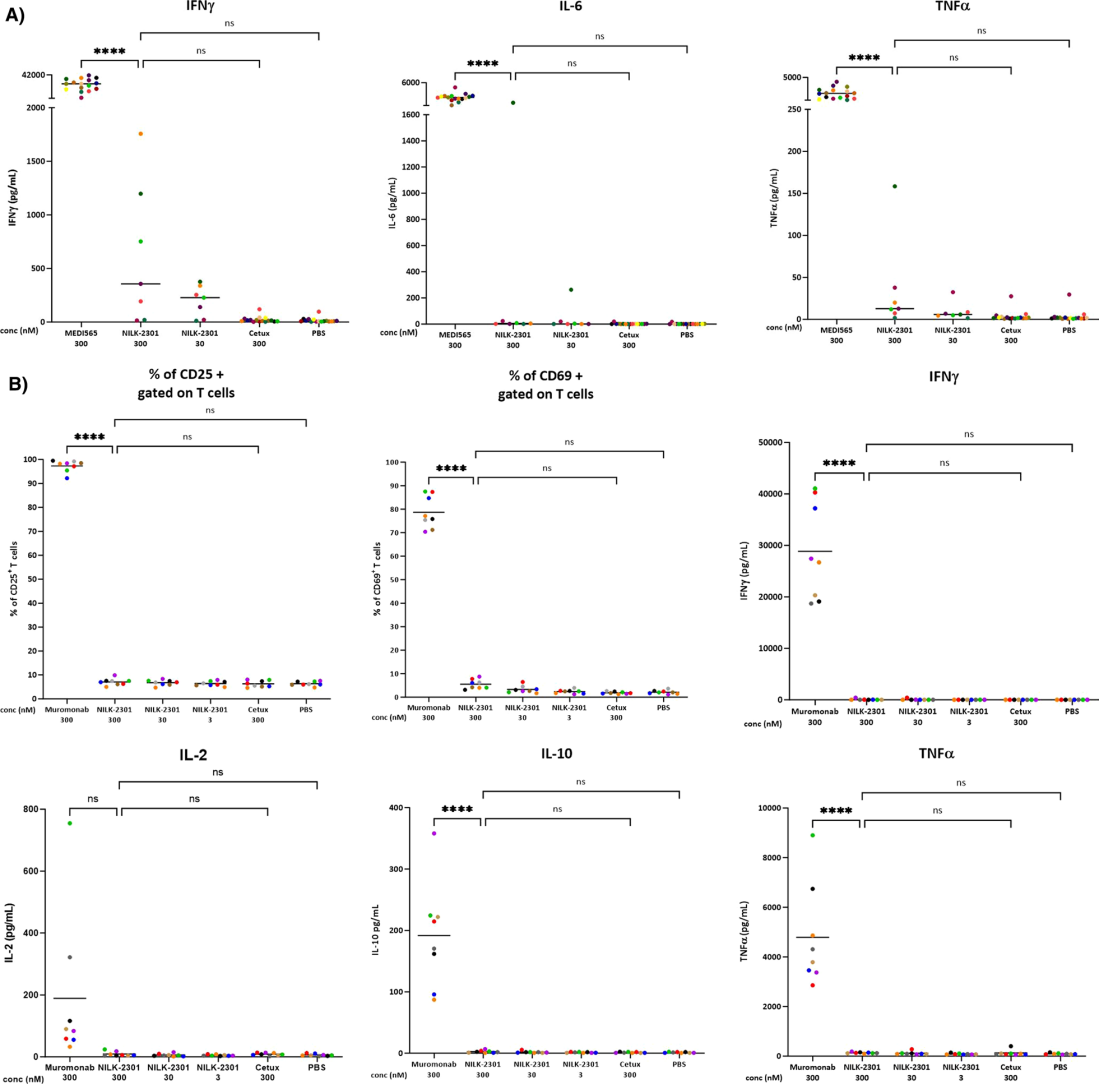
Whole blood and PBMC cytokine release and T-cell activation. **A** Cytokine release from whole blood induced by NILK-2301 following 24 h of incubation. MEDI-565 BiTE and cetuximab (Cetux) were used as control antibodies. Each dot represents the value measured for a given antibody and for a given donor. Measurements obtained with a given donor are pictured with the same color. No significant non-target cell associated cytokine release as compared to control (PBS) was observed for NILK-2301. **B** T-cell activation and cytokine release from healthy donor PBMCs induced by NILK-2301 after 48 h of incubation. Muromonab and cetuximab (Cetux) were used as control antibodies. As for whole blood, no significant non-target cell mediated T-cell activation or cytokine release as compared to control (PBS) or T-cell activation was observed for NILK-2301. Measurements obtained with a given donor are pictured with the same color. Asterisks denote significance to the level of *P < 0.05, **P < 0.01, ***P = 0.001, and ****P < 0.0001. ns, not significant

Using whole blood samples from healthy donors, NILK-2301 (30 nM, 300 nM) did not induce significant cytokine release, similar to PBS and the negative control antibody cetuximab (anti-EGFR mAb) [47] tested at 300 nM (Cetux 300; Fig. 4A). In contrast, a MEDI-565 analog induced significantly higher cytokine release of all cytokines tested, likely due to its cross-reactive binding to CEACAM8 expressed on neutrophils in the blood (data not shown). The observed cytokine release induced by the MEDI-565 analog was consistent with the clinical observations in patients treated with MEDI-565 [35].

We next exposed PBMC samples from healthy donors for 48 h to concentrations of 30 nM and 300 nM of NILK-2301. As MEDI-565 was not potent to induce cytokine release in this assay format due to the absence of neutrophils in PBMCs and the monovalent binding to CD3 by MEDI-565, while the anti-CD3 mAb muronomab was well-established as a positive control in this assay, muronomab was thus selected as a positive control here. NILK-2301 did not induce any significant cytokine release as compared to PBS-control (Fig. 4B). Except for IL-2, all cytokines were released significantly more by muromonab (anti-CD3 mAb OKT3) than by NILK-2301. As observed for cytokines, NILK-2301 at a concentration of 300 nM did not induce significantly higher T-cell activation, as determined by the expression of CD25 and CD69 (Fig. 4B).

Taken together, NILK-2301 does not induce target cell independent T-cell activation in whole blood or using PBMCs from healthy donors. The level of unspecific cytokine release or T-cell activation marker expression is significantly lower as compared to control antibodies. Thus, a favorable toxicity profile of NILK-2301 could be expected.

#### Impact of soluble CEACAM5 on the activity of NILK-2301 in vitro

Because patients affected by CEACAM5-positive malignancies are known to present increased levels of CEACAM5 in circulation due to receptor shedding, we next investigated the impact of soluble CEACAM5 on the activity of NILK-2301.

Binding of NILK-2301 was assessed in the presence of 0.02, 0.05 and 0.1 μg/mL of soluble CEACAM5 for the tumor cell lines SK-CO-1, MKN-45, and H2122. Overall, soluble CEACAM5 did not significantly decrease binding of NILK-2301 to the three investigated cell lines (Additional file 1: Figure S5A–C).

The impact of soluble CEACAM5 on NILK-2301 functionality was assessed by TDCC in the presence of 0.02, 0.05 and 0.1 µg/mL soluble CEACAM5 for the three tumor cell lines mentioned above. Consistent with the binding results, soluble CEACAM5 had no significant impact on TDCC activity (Additional file 1: Figure S5D–F).

#### CEACAM5 receptor occupancy

SK-CO-1, SNU-C1, and LS-174T expressing different levels of CEACAM5 (LS-174T < SNU-C1 < SK-CO-1) were used in a flow cytometry-based assay to determine the CEACAM5 RO mediated by a dose range of NILK-2301.

NILK-2301 binds to all three cell lines in a dose-dependent manner with RO_50_ ranging from 10 to 228 nM (Additional file 1: Figure S6). An inverse correlation between the level of CEACAM5 expression on the target cells and the RO_50_ of NILK-2301 to the target cells was observed.

### In vivo activity

The in vivo anti-tumor activity of NILK-2301 was investigated in two mouse xenograft models using human PBMCs as effector cells and either a colon cancer cell line (LS-174 T) or a pancreatic adenocarcinoma cell line (HPAF-II) as target cells.

#### LS-174T human PBMCs co-grafting model

**Fig. 5:**
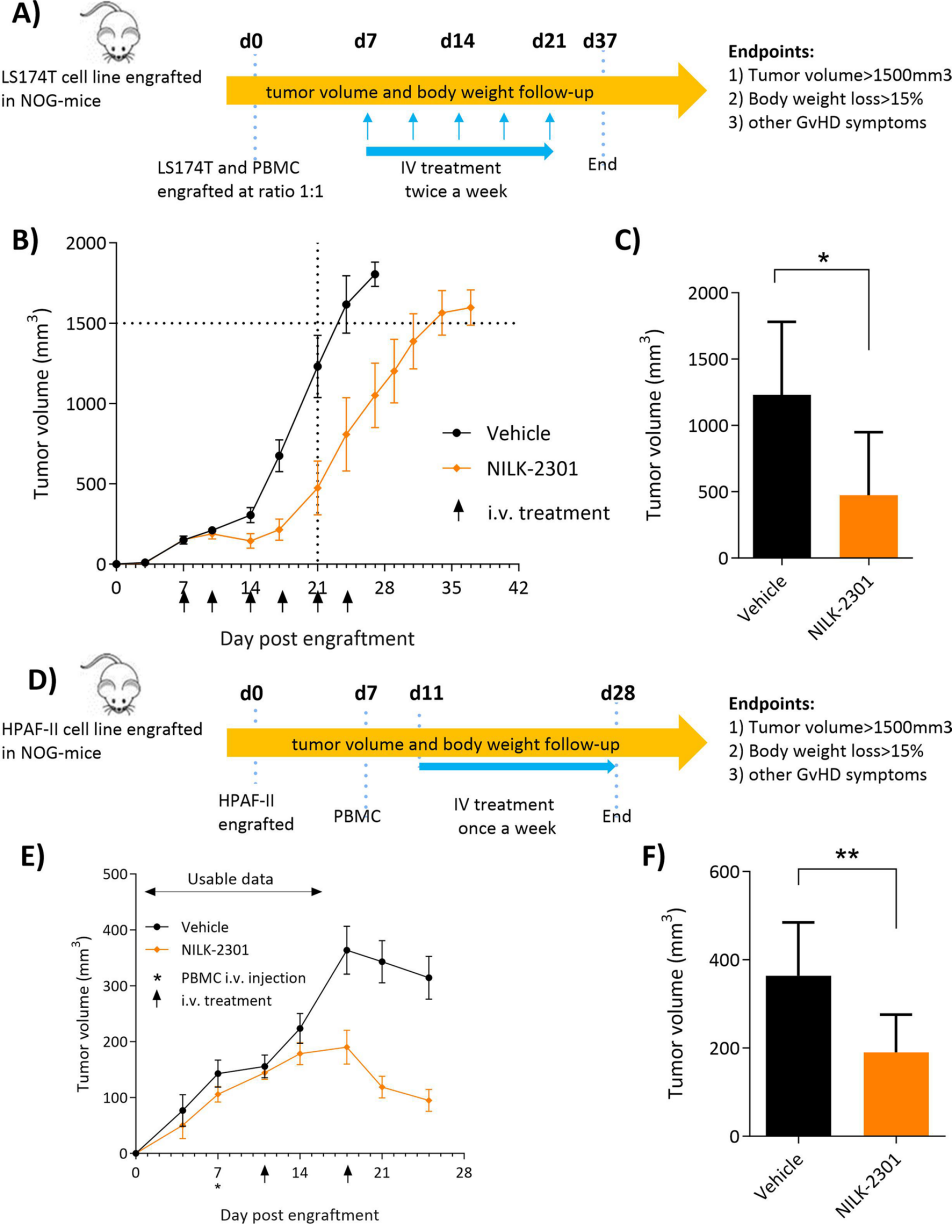
In vivo activity of NILK-2301 in two xenograft models using NOG mice. LS-174 T human PBMCs co-grafting model (upper panel) and HPAF-II PBMCs IV humanized model (lower panel). **A**, **D** Schematic representation of the experimental set up, and **B**, **E** tumor volume over time. **C** Tumor volume at day 21. **F** Tumor volume at day 18. Statistical analysis was performed on tumor volume at day 21 (LS-174 T) or day 18 (HPAF-II), respectively, using Mann–Whitney U test. *P < 0.05 and **P < 0.01. TV, tumor volume; D, day; GvHD, host-versus-graft disease; SC, subcutaneously; IV, intravenously

LS-174T cells and PBMCs were co-grafted SC and treatment was started for all groups at day 7 post co-grafting (Fig. 5A). On day 21, mice in the vehicle group started reaching the ethically pre-defined maximum tumor size endpoint (1500 mm^3^). In contrast, NILK-2301 at 10 mg/kg twice-weekly delayed tumor growth (Fig. 5B). Tumor volume comparison on day 21 reveals a statistically significantly lower tumor burden in mice treated with NILK-2301 compared to vehicle (Fig. 5C).

#### HPAF-II model with IV injected PBMCs

The in vivo efficacy was further confirmed in a model in which HPAF-II cells were grafted SC, followed by IV injection of PBMCs on day 7. When the average tumor volume in mice reached 150 mm^3^, IV treatment was initiated with NILK-2301 (Fig. 5D). As expected from previous experiments, a host-versus-graft reaction leading to spontaneous regression in the control group was observed after day 21. Unbiased data (≤ d18) showed anti-tumor activity for NILK-2301 (Fig. 5E), with a statistically significantly lower tumor burden (*P* < 0.01) measured for NILK-2301 mice compared to vehicle (Fig. 5F).

Taken together, NILK-2301 showed in vivo activity in two independent mouse models using human PBMCs as effector cells either co-grafted SC or injected IV.

### Organoids

Three colon cancer organoid models were selected based on their mRNA CEACAM5 expression level (RNA-sequencing). Cell surface CEACAM5 expression was confirmed by flow cytometry and quantified at 126,000, 47,000, and 38,000 molecules/cell, for organoids HUB-02-C2-019, HUB-02-B2-040 and HUB-02-B2-055, respectively.

**Fig. 6:**
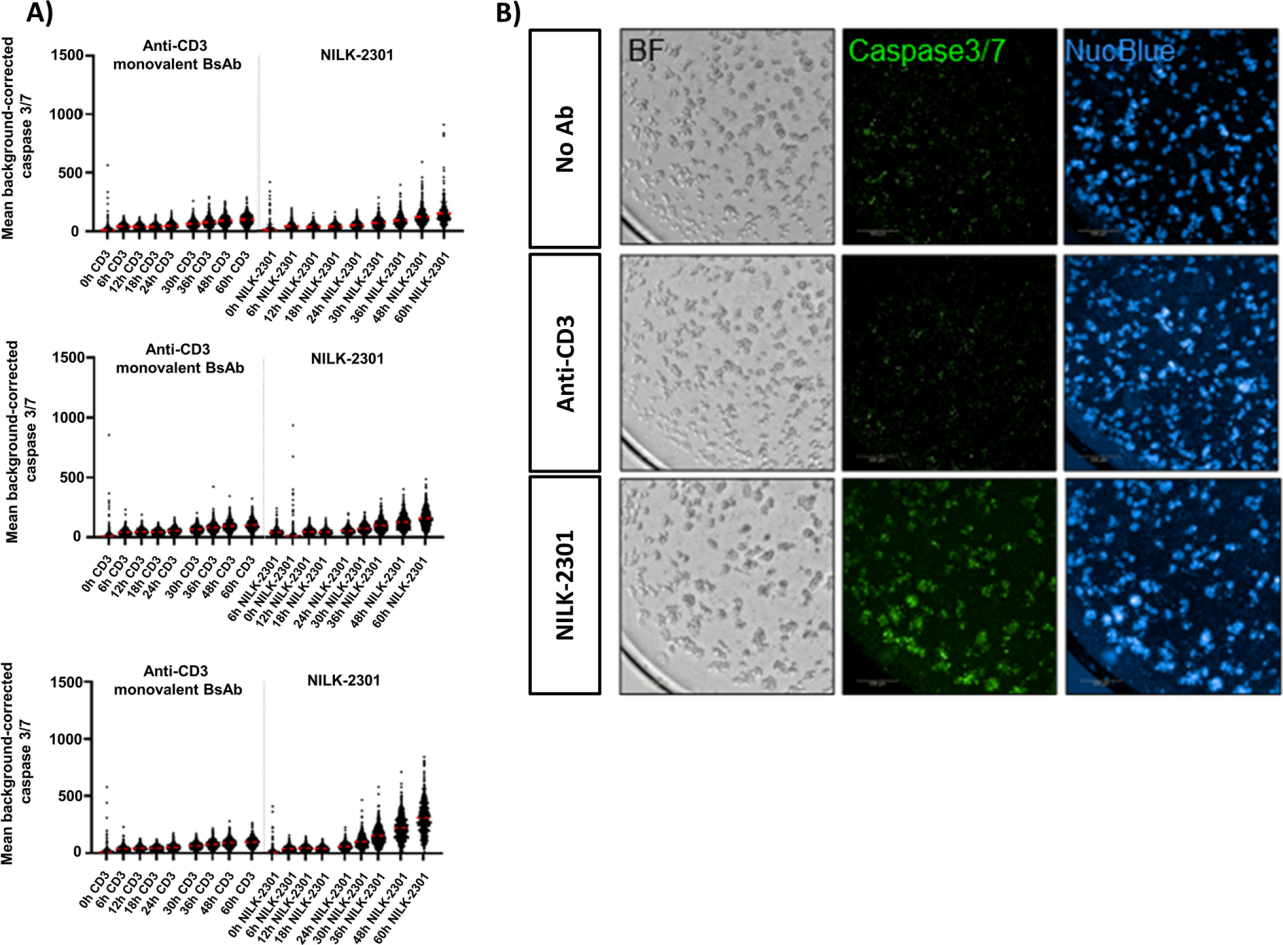
TDCC assay using HUB-02-B2-055 colorectal cancer organoid model. **A** Mean background corrected Caspase3/7 fluorescence intensity from T0 (i.e., 0 h, baseline) to 60 h of co-culture in the presence of anti-CD3 and NILK-2301 plotted per antibody condition for the following concentrations: 0.3 μg/mL (top panel), 1 μg/mL (middle panel) and 5 μg/mL (bottom panel). **B** Representative images at 60 h of co-culture condition with 5 μg/mL NILK-2301. Corresponding mean background corrected Caspase3/7 fluorescence intensity values with their standard deviations are shown in Additional file 1: Table S3. BF, brightfield. Scale bar 500 μm

NILK-2301 at 5 μg/mL resulted in an increased Caspase3/7 signal, i.e., increased apoptosis overtime (exemplary data for HUB-02-B2-055 are shown in Fig. 6A and Additional file 1: Table S3). Of note, 64.2% of the Caspase3/7 signal induced by the apoptosis-inducing agent staurosporine were achieved by NILK-2301 at 5 µg/mL after 60 h of co-culture (310.3 vs. 483.4 relative fluorescence units; Additional file 1: Table S3). Increased apoptosis was visible also from the images taken at the end of the co-culture with 5 μg/mL NILK-2301 (Fig. 6B). No IFN-γ secretion was observed up to the highest tested NILK-2301 concentration of 5 µg/mL (data not shown).

### Primary tumor tissue slices

**Fig. 7:**
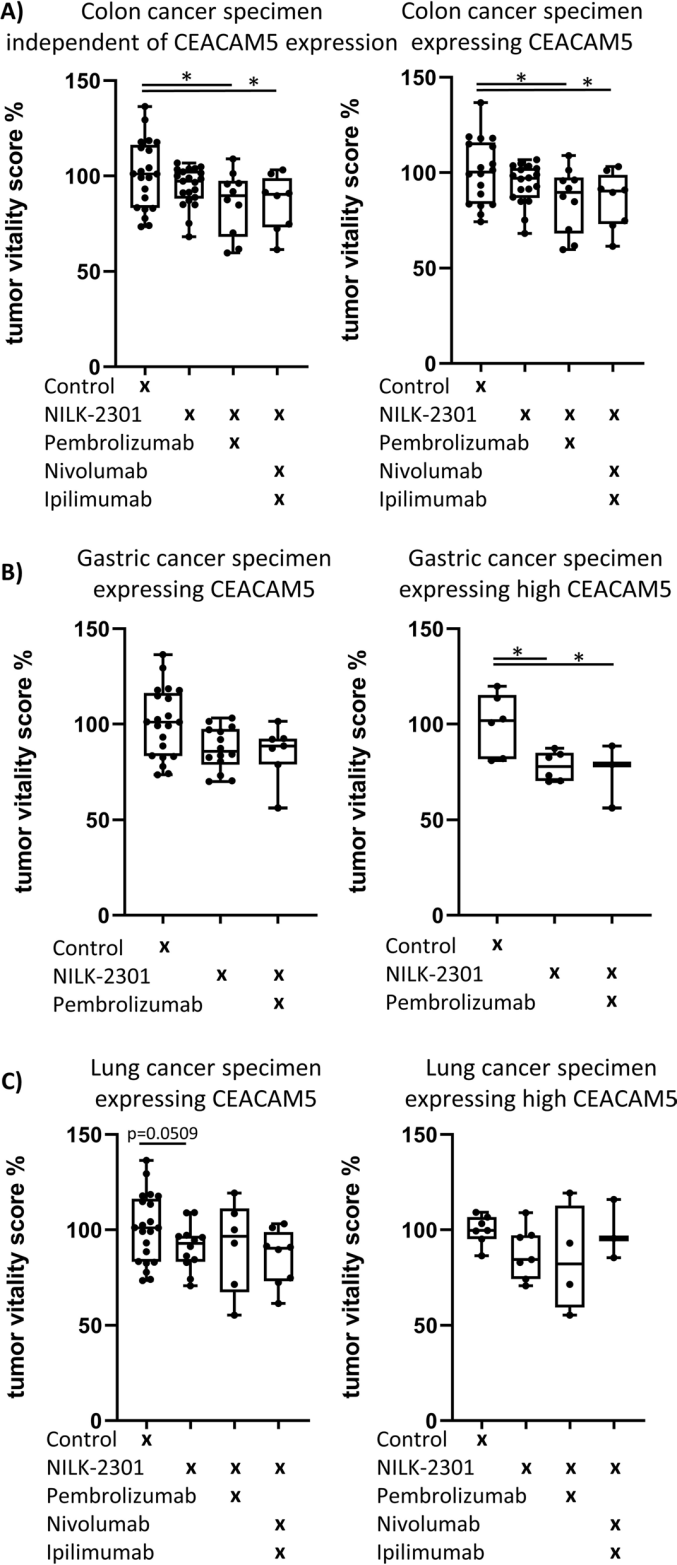

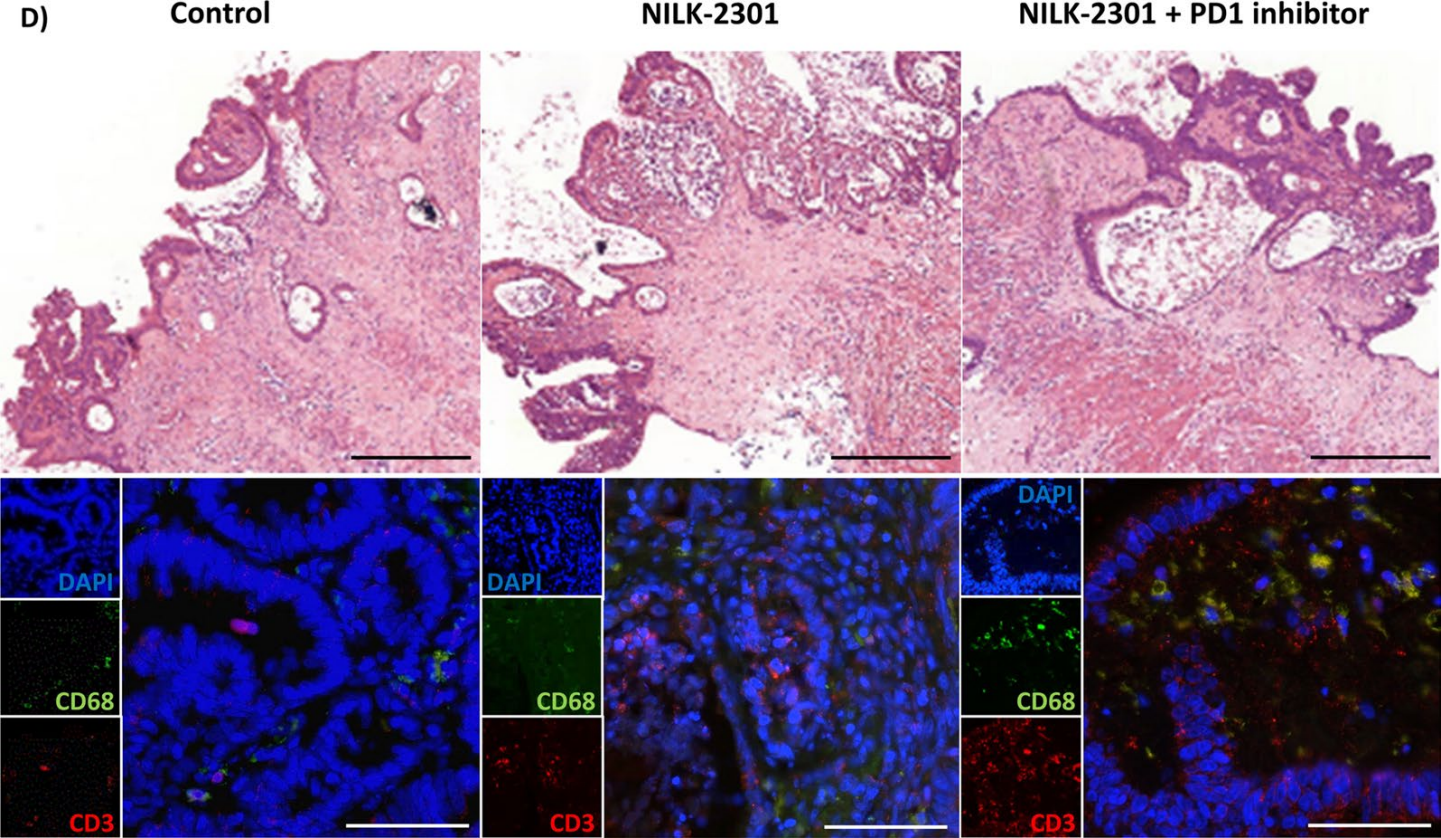
Overall tumor response in PDTC specimens. **A** Samples from all patient specimen and specimen expressing CEACAM5 with colorectal cancer, **B** gastric cancer, and **C** lung cancer. CEACAM5-positive specimens according to immunohistochemistry are shown in the left-hand panels, while specimens expressing high levels of CEACAM5 are shown in the right-hand panels. A summary of the mean values of the normalized tumor responses is given in Additional file 1: Table S4. **D** HE stainings of one representative colon cancer specimen is given, together with the immunofluorescent staining against CD3 and CD68, counterstained with DAPI. HE bar = 250 µm; immunofluorescence bar = 50 µm

NILK-2301 was tested on organotypic PDTCs from surgical resections both in monotherapy as well as in combination with checkpoint inhibitors. Across all PDTC specimens, responses of ≥ 10% were seen in 19/54 samples (35%) treated with NILK-2301 as single agent, 13/28 samples (46%) treated with NILK-2301 + pembrolizumab, and 10/17 samples (59%) treated with the triple combination, i.e., NILK-2301 + nivolumab + ipilimumab (Fig. 7 and Additional file 1: Table S4).

Of all 21 colon cancer specimens, three (14%) did not express CEACAM5 and five (24%) showed a high expression of CEACAM5. The combination of NILK-2301 plus PD-1 inhibitor pembrolizumab showed effects in 6/10 specimens (60%), compared to only 6/ 21 samples (29%) responding to NILK-2301 monotherapy. Of nine PDTCs treated with the triple combination (i.e., NILK-2301 + PD-1 inhibitor nivolumab + CTLA-4 inhibitor ipilimumab), six had a reduction of tumor vitality (67%). Of these, five tissue specimen (83%) showed high CEACAM5 expression. The effect of the triple combination was superior to the double combination in only two PDTCs. Overall, the administration of NILK-2301 plus PD-1 inhibitor mediated the highest efficacy in reducing tumor burden in colon cancer PDTCs.

Fourteen out of sixteen gastric cancer specimens (87.5%) expressed CEACAM5 and were potential candidates for treatment with NILK-2301. Of all gastric cancer specimen investigated, 7/16 (44%) responded to single NILK-2301 treatment and 4/8 (50%) to the combination of NILK-2301 with pembrolizumab. Tumors with high CEACAM5 expression levels (6/14 samples; 43%) responded well to NILK-2301 monotherapy (4/6 specimens; 67%), which was further enhanced in two PDTCs when combined with PD-1 inhibitor pembrolizumab.

The diversity of CEACAM5 expression was the highest in the investigated lung cancer specimen; 6/17 (35%) each did express high or no CEACAM5, respectively. Two of three tissues (67%) with high CEACAM5 expression responded to the triple immunotherapy combination. An effect of the triple combination could also be seen in a tissue with low CEACAM5 as well as in a tissue without CEACAM5 expression according to immunohistochemistry. A larger cohort could be investigated with NILK-2301 monotherapy. Here, 5/11 (45%) CEACAM5-positive PDTCs showed an effect (*P* = 0.0509). While one sample did not respond to any of the conditions, although CEACAM5 was highly expressed, a favorable effect of NILK-2301 was observed in the overall tumor fraction independent of CEACAM5.

Some specimens were stained for CD3 and CD68 to control for the presence of these cellular components regarding tumor response (Fig. 7D). Representative pictures demonstrate CD3-expressing cells in regard to the observed abundancy.

### Pharmacokinetics

NILK-2301 shows cynomolgus anti-CD3 and Fc cross-reactivity but none to CEACAM5. Cynomolgus monkey is therefore a partially relevant species to test PK as well as tolerability and to de-risk potential off-target binding of the anti-CEACAM5 arm of NILK-2301 in vivo*.* The PK profile of NILK-2301 was investigated in male and female cynomolgus monkeys after a single IV injection at 0.5 or 10 mg/kg (groups 1 and 2) or a SC injection at 20 mg/kg (group 3).

**Fig. 8:**
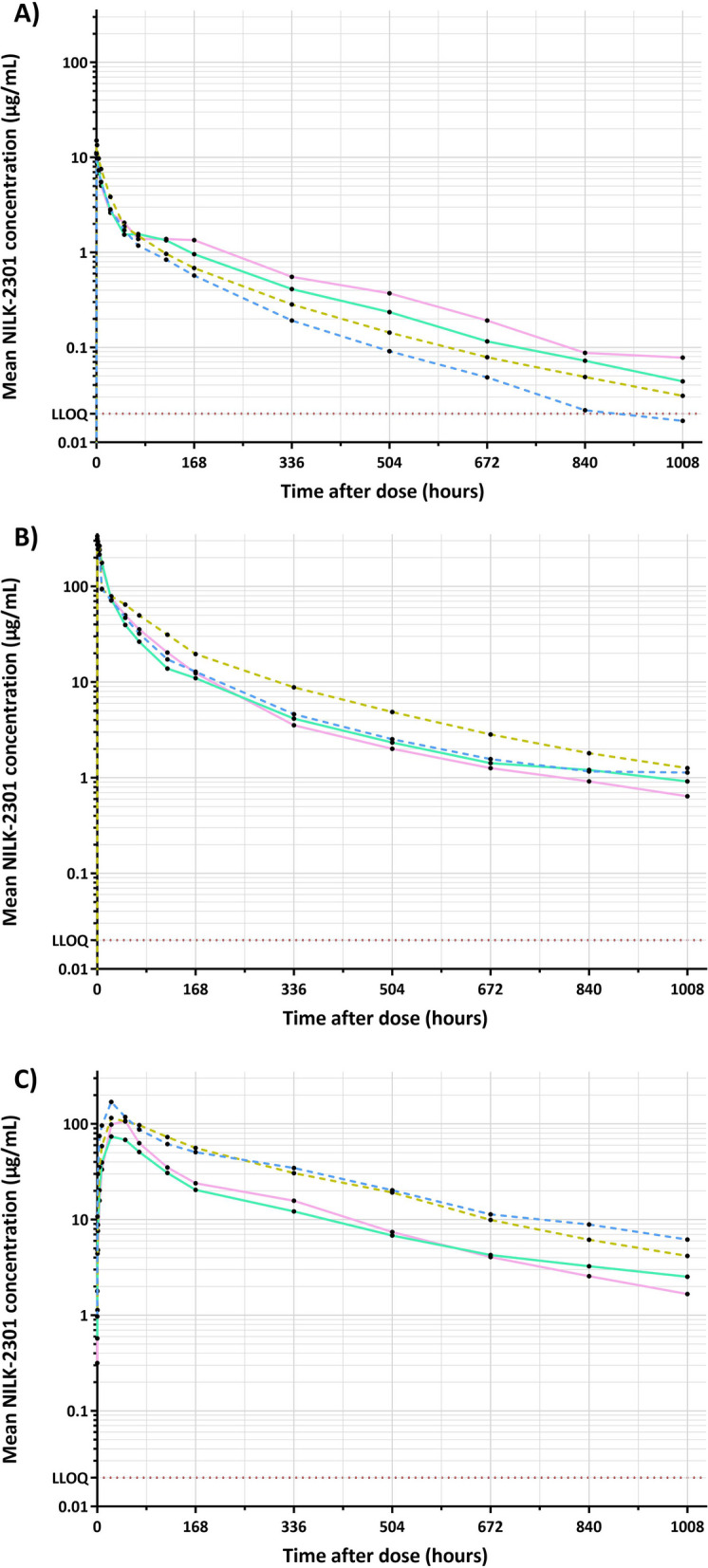
NILK-2301 concentrations versus time in cynomolgus monkeys. Animals received a single IV administration at **A** 0.5 mg/kg, **B** 10 mg/kg, or **C** 20 mg/kg SC, respectively. The four differently colored curves represent the individual animals (two females [solid lines] and two males [dashed lines]). PK parameters are summarized in Additional file 1: Table S5 (IV) and S6 (SC). LLOQ, lower limit of quantification

The concentrations versus time profiles after IV and SC administrations are shown in Fig. 8 with PK parameters summarized in Additional file 1: Table S5 (IV) and S6 (SC).

Following an IV administration at 0.5 mg/kg, concentrations decreased steadily until the last time point. In the two female monkeys, a plateau was observed in the profile. Concentrations after 72 h tended to be lower in males compared to females.

After IV administration at 10 mg/kg, concentrations decreased steadily till the last time point and tended to be slightly higher in one male monkey.

Following SC administration at 20 mg/kg, quantifiable NILK-2301 concentrations were observed at pre-dose (since the same monkeys participated in both groups 2 and 3) until the last time point. The absorption phase was variable with the peak concentration varying between 73.8 and 170 μg/mL at 24–48 h post-dose. After reaching the peak, concentrations gradually decreased with relatively similar elimination phase between the monkeys. Concentrations were slightly higher in males compared to females.

Individual bioavailability (between groups IV 10 mg/kg and SC 20 mg/kg) ranged from 75.5 to 172%, and bioavailability calculated on median values was 112% and 110% for IV at 0.5 and 10 mg/kg respectively. No trend between males and females was observed after IV administrations while AUCs tended to be higher and CL to be lower in males compared with females after SC administration. The predicted half-life in humans is 13.1 days.

PK data were confirmed in Tg32 human FcRn mice. NILK-2301 PK seemed to be dose-proportional for *C*_max_ and AUC_last_ for the 0.5 and 10 mg/kg dose levels (data not shown). CL values were 0.35–0.44 mL/h/kg and seemed consistent with the range reported previously for 17 monoclonal antibodies in wild-type mice (0.13–2.19 mL/h/kg, median: 0.36 mL/h/kg) [48].

### Tolerability

Doses of 0.5 and 10 mg/kg IV and 20 mg/kg SC were well-tolerated over the 6-week observation period in cynomolgus monkeys and did not lead to any in-life findings or toxicological changes at the injection site, in body weights, food consumption or anatomical pathology. In addition, the 0.5 and 10 mg/kg groups, also evaluated for safety pharmacology, immunophenotyping, clinical pharmacology changes, revealed no toxicological changes in any of these parameters.

Likewise, NILK-2301 was well-tolerated in human FcRn Tg32 mice; no clinical signs, no weight loss, or mortality were observed following injections of 0.5 and 10 mg/kg NILK-2301, respectively.

## Discussion

Here, we describe a novel CEACAM5xCD3 BsAb, termed NILK-2301, constructed using the κλ body platform. This format is assembled by co-expressing one heavy chain and two different light chains, one κ and one λ, resulting in a BsAb that fully retains the sequence and architecture of human IgG antibodies [39]. The κλ-format presumably conveys low antigenicity and concomitantly lower potential for induction of neutralizing ADAs. Besides, NILK-2301 contains additional inbuilt characteristics to limit unspecific activation and potential side effects. The molecule first binds with higher affinity to tumor cells (*K*_D_≈5 nM) as compared to T-cells (*K*_D_≈100 nM), limiting unspecific T-cell activation. Secondly, Fc-mediated effector functions are abrogated by introduction of three mutations (“LALAPA”) [44–46], to convey a favorable safety profile in patients due to the lack of binding to activating FcγRs (i.e., no CD3 cross-linking). The binding to the FcRn is however maintained, to retain the typical half-life of IgGs in circulation.

Simultaneous binding of NILK-2301 to CEACAM5-positive tumor cells and CD3ε-expressing T-cells leads to T-cell activation, proliferation, and secretion of cytokines and cytotoxic enzymes, ultimately leading to tumor cell killing. Overall, NILK-2301 showed promising in vitro and in vivo activity, including data from 3D models, i.e., PDTCs from patients with colon, gastric or lung cancer and colorectal cancer organoids, which have been shown to be able to overcome the various limitations of standard in vitro 2D cultures and mouse in vivo models [49]. The tissue response rate in PDTCs expressing CEACAM5 is notable, given the short incubation time compared to duration of patient treatment and the effect size in this clinically relevant model system. Sixty percent of cultures from patients with colon cancer responded to NILK-2301 plus pembrolizumab and about 45% of the tissue cultures of gastric cancer and lung cancer specimens, respectively, responded to NILK-2301 monotherapy. The effect of pembrolizumab alone was not investigated in the present study, however, the combined tissue response rate of 60% in colon cancer PDTCs for NILK-2301 plus pembrolizumab in our study exceeded the overall response rate of 43.8% found for pembrolizumab monotherapy in microsatellite-instability-high advanced colorectal cancer patients [50]. Previous results in gastric cancer cultures demonstrated that the effect of the PD-1 inhibitor nivolumab can be enhanced when combined with immune activators (e.g., CD3/CD28 T-lymphocyte activator ImmunoCult™^)^, resulting in similar effect sizes to those found in the present study [51]. For comparison, the use of oxaliplatin at unphysiologically high concentrations (i.e., 100 μM) was equally effective as NILK-2301 monotherapy [51]. NILK-2301 showed also positive effects on the overall tumor fraction of lung cancer samples, independent of CEACAM5 expression and should be further evaluated alone and in combination with immune checkpoint inhibition.

Likewise, NILK-2301 induced increased Caspase3/7 signal, i.e., increased apoptosis, in colorectal cancer organoid models. Quantification of Caspase3/7 signal appeared as suitable surrogate readout for T-cell mediated killing and activation in co-cultures of organoids and PBMCs. However, optimizations of the killing readout for future analyses might be required, given that a non-specific Caspase3/7 increase across all conditions has been observed using different organoid models (data not shown), potentially due to alloreactivity effects and/or experimental conditions. When using an allogeneic PBMC donor in a co-culture assay for the first time it is thus important to assess its compatibility with the selected organoid model beforehand to avoid excessive non-specific organoid killing in the absence of antibodies, reducing the assay window.

In vitro studies with NILK-2301 indicated that no relevant animal species exists for nonclinical safety studies because the anti-CEACAM5 arm is not cross-reactive with cynomolgus and no CEACAM5 orthologs exist in rodents or other species commonly used in nonclinical safety evaluations [52]. We and others have been unable to generate CEACAM5-specific relevant cynomolgus surrogate mAbs, and murine transgenic humanCEACAM5 x humanCD3 mice have been shown as inappropriate too [53]. Notwithstanding, NILK-2301 shows cynomolgus anti-CD3 and Fc cross-reactivity. Cynomolgus monkey is thus a partially relevant species to test PK and tolerability of NILK-2301 in vivo*,* in which the anti-CD3 and Fc-binding as well as the potential off-target binding of the anti-CEACAM5 arm could be de-risked. In this model, the well-tolerated dose of NILK-2301 was set at 10 mg/kg, i.e., the highest IV dose administered. Also, in human FcRn Tg32 mice, no FcRn-related or unspecific toxicity or treatment-related changes were observed. While cynomolgus and Tg32 mice were deemed relevant for hazard identification, given the lack of full cross-reactivity (and thus pharmacological activity), no standard toxicity studies were considered justified in these species.

Different molecules targeting CEACAM5 have already been tested or are currently in clinical trials [32–38]. Of these, CEACAM5-targeting T-cell engagers conveying the same mechanism of action are closest to NILK-2301 and can thus be taken as reference. These include the MEDI-565 BiTE (NCT01284231, NCT02291614) [29, 35, 37] as well as cibisatamab (NCT02324257, NCT02650713, NCT04826003) [28, 34, 54, 55] and RO7172508 (RG6123; NCT03539484) [38] based on the so-called 2:1 TCB format. BiTEs contain one single chain variable fragment (scFv) with specificity for a tumor-associated antigen molecularly fused to another scFv with specificity for CD3 on T-cells; the molecular weight is approximately 54 kDa (twice the 27 kDa for scFv). MEDI-565 binds with a comparable K_D_ of 6 nM to the A2 domain of CEACAM5 (the same to which NILK-2301 binds) [29, 56]. One of the main disadvantages of BiTEs due to their format is the fast clearance and short half-life, requiring continuous infusion, which has limited their use in routine clinical practice [57]. In contrast, cibisatamab (molecular weight approximately 192.78 kD) is an IgG-based TCB that incorporates bivalent binding to CEACAM5, a head-to-tail fusion of CEACAM5- and CD3e-binding Fab domains as well as an engineered Fc region with completely abolished binding to FcγRs and C1q [28]. The molecule binds to the B3 domain of CEACAM5 with an affinity of K_D_ 5–16 nM (i.e., comparable to NILK-2301). RO7172508 (RG6123) is likewise of 2:1 format with a humanized CEACAM5 binder to the A3-B3 domain of CEACAM5 with very high monovalent affinity (K_D_ 90 pM). Signs of clinical activity, e.g., reaching stable disease or increase in cytokine levels as pharmacodynamics marker, have been seen with all three molecules; however, formal objective response could only be observed with cibisatamab. Partial responses were seen with monotherapy and further enhanced in combination with checkpoint inhibition (atezolizumab), with 6% vs. 18% in patients receiving ≥ 60 mg cibisatamab flat dose [34]. CEACAM5-targeting T-cell engagers showed an acceptable and manageable safety profile with GI toxicity (on-target) being the main reported adverse event and dose-limiting toxicity. ADAs were found in approximately half of patients for all three molecules, and such ADA-mediated loss of exposure limited clinical efficacy and, for example in the case of MEDI-565 [37], precluded the definition of a therapeutic window, which may be due to the artificial formats (i.e., BiTE or especially 2:1 format).

Based on these findings, it can be assumed that a BsAb with presumably lower potential of ADA-formation, such as the so-called κλ body format used here [39], for which clinical evidence is given by ongoing clinical trials involving other BsAbs based on the same κλ body format, i.e., the CD19xCD47 BsAb NI-1701 (TG-1801) [58] and MSLNxCD47 (NI-1801) [59], could be of significant therapeutic potential.

Given the mechanism of action of NILK-2301 compared with other approved anti-cancer agents and the presumably non-overlapping toxicity profile, it is anticipated that NILK-2301 will combine favorably with other treatment modalities, e.g., immune checkpoint inhibitors [34], co-stimulatory agents like tumor-targeted 4-1BB agonists or CD28-BsAbs [60–62], or CD47-targeting BsAbs [63], in future clinical studies.

## Conclusion

In conclusion, NILK-2301 combines promising preclinical activity and safety with format-associated lower probability of ADA-generation and PK consistent with IV or SC applications, e.g., every one or two weeks. NILK-2301 will enter clinical testing at the end of 2023 in patients with advanced colorectal adenocarcinoma.

### Supplementary Information

**Additional file 1.** Data supplement.

## Data Availability

All data generated or analyzed during this study are included in this published article and its supplementary information file.

## References

[1] Weiner GJ (2015). Building better monoclonal antibody-based therapeutics. Nat Rev Cancer.

[2] Sharma P, Allison JP (2015). The future of immune checkpoint therapy. Science.

[3] Matlung HL, Szilagyi K, Barclay NA, van den Berg TK (2017). The CD47-SIRPalpha signaling axis as an innate immune checkpoint in cancer. Immunol Rev.

[4] Tapia-Galisteo A, Álvarez-Vallina L, Sanz L (2023). Bi- and trispecific immune cell engagers for immunotherapy of hematological malignancies. J Hematol Oncol.

[5] Guha P, Heatherton KR, O’Connell KP, Alexander IS, Katz SC (2022). Assessing the future of solid tumor immunotherapy. Biomedicines.

[6] Ma W, Xue R, Zhu Z (2023). Increasing cure rates of solid tumors by immune checkpoint inhibitors. Exp Hematol Oncol.

[7] Mitra A, Barua A, Huang L, Ganguly S, Feng Q, He B (2023). From bench to bedside: the history and progress of CAR T cell therapy. Front Immunol.

[8] Mendoza-Valderrey A, Alvarez M, De Maria A, Margolin K, Melero I, Ascierto ML (2022). Next generation immuno-oncology strategies: unleashing NK cells activity. Cells.

[9] Mak TWaS, Mary E (2006). T cell activation. The immune response.

[10] Mir MA. Chapter 1 - Introduction to Costimulation and Costimulatory Molecules. In: Mir MA, ed. Developing costimulatory molecules for immunotherapy of diseases: academic press; 2015:1–43.

[11] Schuster SJ, Bishop MR, Tam CS (2018). Tisagenlecleucel in adult relapsed or refractory diffuse large B-cell lymphoma. N Engl J Med.

[12] Munshi NC, Anderson LD, Shah N (2021). Idecabtagene Vicleucel in Relapsed and Refractory Multiple Myeloma. N Engl J Med.

[13] Locke FL, Miklos DB, Jacobson CA (2021). Axicabtagene ciloleucel as second-line therapy for large B-cell lymphoma. N Engl J Med.

[14] Martin T, Usmani SZ, Berdeja JG (2023). Ciltacabtagene autoleucel, an anti-B-cell maturation antigen chimeric antigen receptor T-cell therapy, for relapsed/refractory multiple myeloma: CARTITUDE-1 2-year follow-up. J Clin Oncol.

[15] Seckinger A, Delgado JA, Moser S (2017). Target expression, generation, preclinical activity, and pharmacokinetics of the BCMA-T cell bispecific antibody EM801 for multiple myeloma treatment. Cancer Cell.

[16] Wong SW, Bar N, Paris L (2022). Alnuctamab (ALNUC; BMS-986349; CC-93269), a B-cell maturation antigen (BCMA) x CD3 T-cell engager (TCE), in patients (pts) with relapsed/refractory multiple myeloma (RRMM): results from a phase 1 first-in-human clinical study. Blood.

[17] Usmani SZ, Garfall AL, van de Donk N (2021). Teclistamab, a B-cell maturation antigen x CD3 bispecific antibody, in patients with relapsed or refractory multiple myeloma (MajesTEC-1): a multicentre, open-label, single-arm, phase 1 study. Lancet.

[18] Hutchings M, Morschhauser F, Iacoboni G (2021). Glofitamab, a novel, bivalent CD20-targeting T-cell-engaging bispecific antibody, induces durable complete remissions in relapsed or refractory B-cell lymphoma: a Phase I Trial. J Clin Oncol.

[19] Gold P, Freedman SO (1965). Specific carcinoembryonic antigens of the human digestive system. J Exp Med.

[20] Gold P, Freedman SO (1965). Demonstration of tumor-specific antigens in human colonic carcinomata by immunological tolerance and absorption techniques. J Exp Med.

[21] Beauchemin N, Arabzadeh A (2013). Carcinoembryonic antigen-related cell adhesion molecules (CEACAMs) in cancer progression and metastasis. Cancer Metastasis Rev.

[22] Yamamoto Y, Hirakawa E, Mori S, Hamada Y, Kawaguchi N, Matsuura N (2005). Cleavage of carcinoembryonic antigen induces metastatic potential in colorectal carcinoma. Biochem Biophys Res Commun.

[23] Sack TL, Gum JR, Low MG, Kim YS (1988). Release of carcinoembryonic antigen from human colon cancer cells by phosphatidylinositol-specific phospholipase C. J Clin Investig.

[24] Naghibalhossaini F, Ebadi P (2006). Evidence for CEA release from human colon cancer cells by an endogenous GPI-PLD enzyme. Cancer Lett.

[25] Pakdel A, Naghibalhossaini F, Mokarram P, Jaberipour M, Hosseini A (2012). Regulation of carcinoembryonic antigen release from colorectal cancer cells. Mol Biol Rep.

[26] Hammarstrom S (1999). The carcinoembryonic antigen (CEA) family: structures, suggested functions and expression in normal and malignant tissues. Semin Cancer Biol.

[27] Kuespert K, Pils S, Hauck CR (2006). CEACAMs: their role in physiology and pathophysiology. Curr Opin Cell Biol.

[28] Bacac M, Fauti T, Sam J (2016). A novel carcinoembryonic antigen T-Cell bispecific antibody (CEA TCB) for the treatment of solid tumors. Clin Cancer Res.

[29] Oberst MD, Fuhrmann S, Mulgrew K (2014). CEA/CD3 bispecific antibody MEDI-565/AMG 211 activation of T cells and subsequent killing of human tumors is independent of mutations commonly found in colorectal adenocarcinomas. MAbs.

[30] Boucher D, Cournoyer D, Stanners CP, Fuks A (1989). Studies on the control of gene expression of the carcinoembryonic antigen family in human tissue. Cancer Res.

[31] Decary S, Berne PF, Nicolazzi C (2020). Preclinical activity of SAR408701: a novel anti-CEACAM5-maytansinoid antibody-drug conjugate for the treatment of CEACAM5-positive epithelial tumors. Clin Cancer Res.

[32] Liersch T, Meller J, Kulle B (2005). Phase II trial of carcinoembryonic antigen radioimmunotherapy with 131I-labetuzumab after salvage resection of colorectal metastases in the liver: five-year safety and efficacy results. J Clin Oncol.

[33] Dotan E, Cohen SJ, Starodub AN (2017). Phase I/II Trial of Labetuzumab Govitecan (Anti-CEACAM5/SN-38 antibody-drug conjugate) in patients with refractory or relapsing metastatic colorectal cancer. J Clin Oncol.

[34] Tabernero J, Melero I, Ros W, et al. Phase Ia and Ib studies of the novel carcinoembryonic antigen (CEA) T-cell bispecific (CEA CD3 TCB) antibody as a single agent and in combination with atezolizumab: Preliminary efficacy and safety in patients with metastatic colorectal cancer (mCRC). *Journal of Clinical Oncology*. 2017;35(15_suppl):3002–3002.

[35] Pishvaian M, Morse MA, McDevitt J (2016). Phase 1 dose escalation study of MEDI-565, a bispecific T-cell engager that targets human carcinoembryonic antigen, in patients with advanced gastrointestinal adenocarcinomas. Clin Colorectal Cancer.

[36] Gazzah A, Bedard PL, Hierro C (2022). Safety, pharmacokinetics, and antitumor activity of the anti-CEACAM5-DM4 antibody–drug conjugate tusamitamab ravtansine (SAR408701) in patients with advanced solid tumors: first-in-human dose-escalation study. Ann Oncol.

[37] Moek KL, Fiedler WM, von Einem JC (2018). Phase I study of AMG 211/MEDI-565 administered as continuous intravenous infusion (cIV) for relapsed/refractory gastrointestinal (GI) adenocarcinoma. Ann Oncol.

[38] NCT03539484. A study of RO7172508 in patients with locally advanced and/or metastatic CEA-Positive solid tumors. 2018;2023. https://www.clinicaltrials.gov/ct2/show/results/NCT03539484?cond=RO7172508&draw=2&rank=1

[39] Fischer N, Elson G, Magistrelli G (2015). Exploiting light chains for the scalable generation and platform purification of native human bispecific IgG. Nat Commun.

[40] Hatterer E, Barba L, Noraz N (2019). Co-engaging CD47 and CD19 with a bispecific antibody abrogates B-cell receptor/CD19 association leading to impaired B-cell proliferation. MAbs.

[41] Nouveau L, Buatois V, Cons L (2021). Immunological analysis of the murine anti-CD3-induced cytokine release syndrome model and therapeutic efficacy of anti-cytokine antibodies. Eur J Immunol.

[42] Sonnichsen R, Hennig L, Blaschke V (2018). Individual susceptibility analysis using patient-derived slice cultures of colorectal carcinoma. Clin Colorectal Cancer.

[43] Pessano S, Oettgen H, Bhan AK, Terhorst C (1985). The T3/T cell receptor complex: antigenic distinction between the two 20-kd T3 (T3-delta and T3-epsilon) subunits. EMBO J.

[44] Tamm A, Schmidt RE (1997). IgG binding sites on human Fc gamma receptors. Int Rev Immunol.

[45] Lund J, Pound JD, Jones PT (1992). Multiple binding sites on the CH2 domain of IgG for mouse Fc gamma R11. Mol Immunol.

[46] Shields RL, Namenuk AK, Hong K (2001). High resolution mapping of the binding site on human IgG1 for Fc gamma RI, Fc gamma RII, Fc gamma RIII, and FcRn and design of IgG1 variants with improved binding to the Fc gamma R. J Biol Chem.

[47] Bailey L, Moreno L, Manigold T (2013). A simple whole blood bioassay detects cytokine responses to anti-CD28SA and anti-CD52 antibodies. J Pharmacol Toxicol Methods.

[48] Avery LB, Wang M, Kavosi MS (2016). Utility of a human FcRn transgenic mouse model in drug discovery for early assessment and prediction of human pharmacokinetics of monoclonal antibodies. MAbs.

[49] Zhou Z, Pang Y, Ji J (2023). Harnessing 3D in vitro systems to model immune responses to solid tumours: a step towards improving and creating personalized immunotherapies. Nat Rev Immunol.

[50] André T, Shiu K-K, Kim TW (2020). Pembrolizumab in microsatellite-instability–high advanced colorectal cancer. N Engl J Med.

[51] Husstegge M, Hoang NA, Rebstock J (2021). PD-1 inhibition in patient derived tissue cultures of human gastric and gastroesophageal adenocarcinoma. Oncoimmunology.

[52] Kammerer R, Zimmermann W (2010). Coevolution of activating and inhibitory receptors within mammalian carcinoembryonic antigen families. BMC Biol.

[53] Dudal S, Hinton H, Giusti AM (2016). Application of a MABEL approach for a T-cell-bispecific monoclonal antibody: CEA TCB. J Immunother.

[54] Bacac M, Klein C, Umana P (2016). CEA TCB: A novel head-to-tail 2:1 T cell bispecific antibody for treatment of CEA-positive solid tumors. OncoImmunology.

[55] Melero I, Segal NH, Suarez JMS, et al. Pharmacokinetics (PK) and pharmacodynamics (PD) of a novel carcinoembryonic antigen (CEA) T-cell bispecific antibody (CEA CD3 TCB) for the treatment of CEA-expressing solid tumors. J Clinical Oncology. 2017;35(15_suppl):2549–2549.

[56] Choudary S, Parupudi A (Medimmune LLC). Formulations of bispecific antibodies; patent application PCT/US2015/047843 (WO2016036678A1); 2015.

[57] Tian Z, Liu M, Zhang Y, Wang X (2021). Bispecific T cell engagers: an emerging therapy for management of hematologic malignancies. J Hematol Oncol.

[58] Hawkes E, Lewis KL, Wong Doo N (2022). First-in-Human (FIH) Study of the Fully-Human Kappa-Lambda CD19/CD47 Bispecific Antibody TG-1801 in Patients (pts) with B-Cell Lymphoma. Blood.

[59] Romano E, Medioni J, Rouge TDLM (2022). 707 A Phase 1, open-label, dose finding study of NI-1801, a bispecific mesothelin x CD47 engaging antibody, in patients with mesothelin expressing solid cancers. J Immunother Cancer.

[60] Claus C, Ferrara C, Xu W (2019). Tumor-targeted 4–1BB agonists for combination with T cell bispecific antibodies as off-the-shelf therapy. Sci Transl Med.

[61] Seckinger AaM, Moine V, Nouveau L, Daubeuf B, Buatois V, Gueneau F, Ravn U, Masternak K, Poitevin Y, Magistrelli G, Malinge P, Shang L, Fischer N, Strein K, Ferlin WG, Hose D. Novel CEAxCD3 (NILK-2301) and CEAxCD28 (NILK-3301) kl bispecific antibodies for next generation immunotherapy of CEA-expressing cancer. ESMO Congress 2022. Ann. Oncol. 2022;33, S888.

[62] Skokos D, Waite JC, Haber L (2020). A class of costimulatory CD28-bispecific antibodies that enhance the antitumor activity of CD3-bispecific antibodies. Sci Transl Medine..

[63] Seckinger A, Buatois V, Moine V (2022). Novel CEAxCD47 (NILK-2401) and CEAxCD3 (NILK-2301) kl bispecific antibodies for multimodal immunotherapy of CEA-expressing solid cancer. J Immunother Cancer.

