# Supplementary material for: Development and characterization of NILK-2301, a novel CEACAM5xCD3 κλ bispecific antibody for immunotherapy of CEACAM5-expressing cancers
Source: J Hematol Oncol. 2023 Dec 12;16:117. doi: 10.1186/s13045-023-01516-3 (PMC10717981; doi:10.1186/s13045-023-01516-3)
Supplement: Supplementary file 1 — Additional file 1. Data supplement. [file 13045_2023_1516_MOESM1_ESM.docx]

**DATA Supplement**

Supplementary Figures


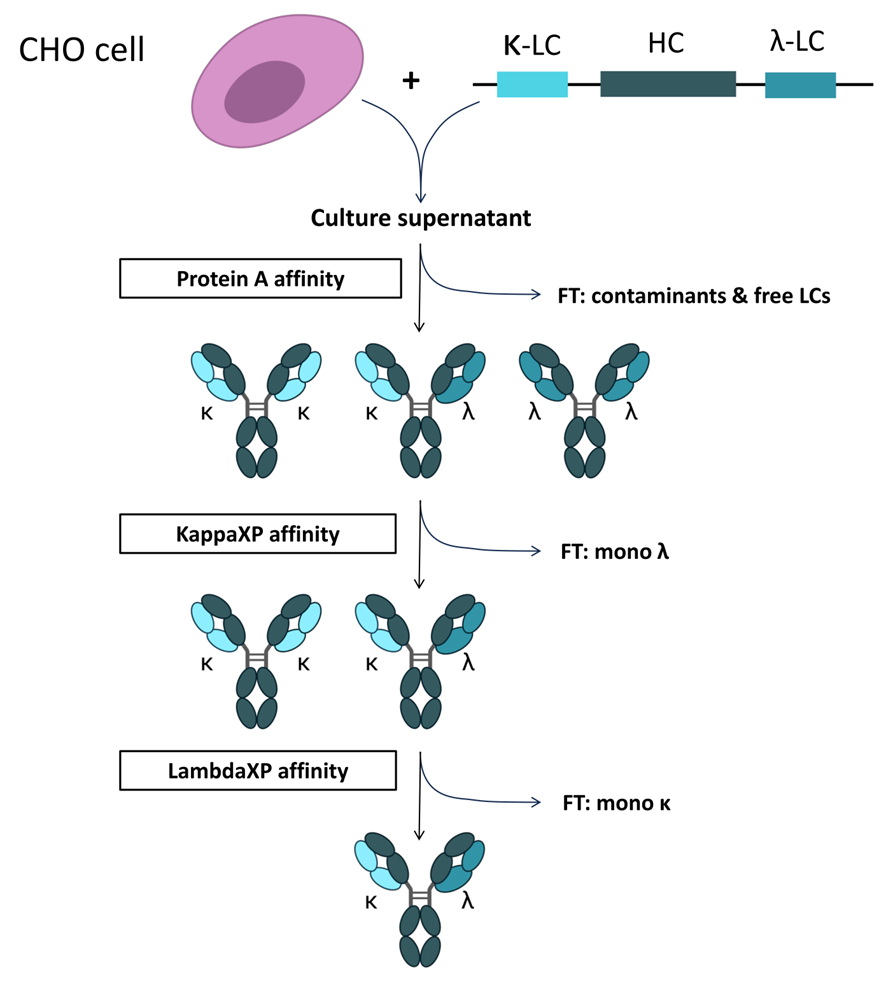


**Supplementary Figure S1. Production and purification of NILK-2301.** NILK-2301 was produced by transfecting a plasmid encoding for the three antibody chains into a CHO host cell line. A three-step affinity purification process allows the isolation of the κλ body from the culture supernatant. CHO, Chinese Hamster Ovary; LC, light chain, HC: heavy chain, FT: flow-through.

**
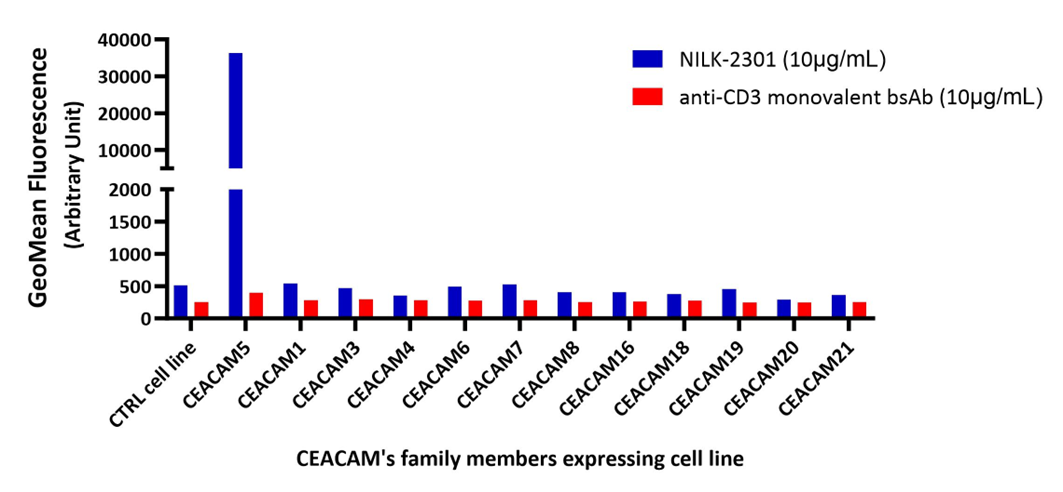
**

**Supplementary Figure S2.** **Binding to different CEACAMs transiently expressed on PEAK cells.** The absence of cross-reactivity of NILK-2301 vs. anti-CD3 monovalent bispecific antibody control toward other CEACAM proteins was confirmed by flow cytometric binding assay using PEAK cells transfected with different members of the CEACAM family. Non-transfected PEAK cells were used as negative control (CTRL). See also Supplementary Table S1. BsAb, bispecific antibody.


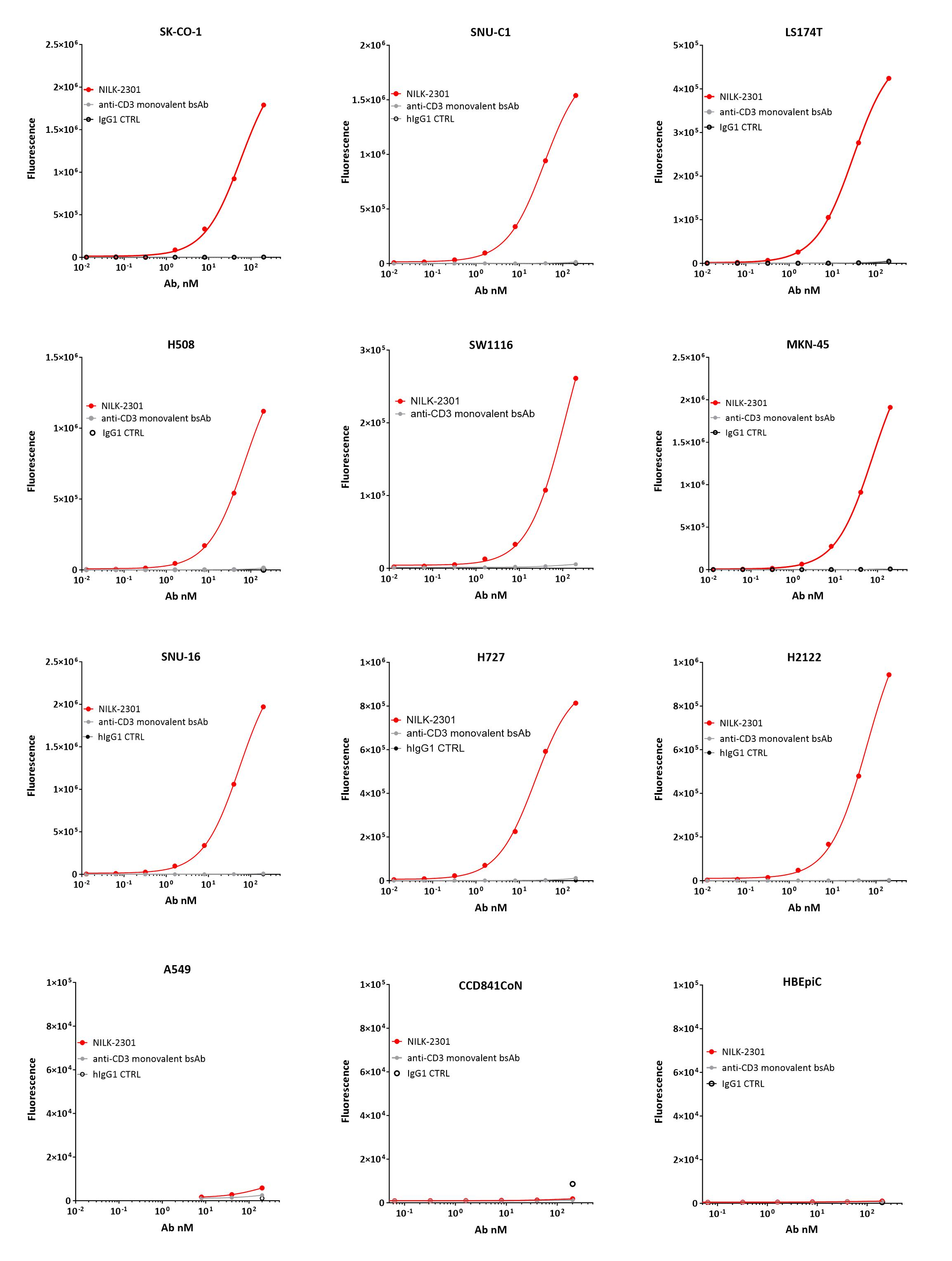


**Supplementary Figure S3. Binding of NILK-2301 to CEACAM5-positive cell lines.** SK-CO-1, SNU-C1, LS-174T, H508, SW1116, MKN-45, SNU-16, H727, and H2122. Absence of binding was observed for CEACAM5-negative cell line A549 and to CCD841CoN, isolated from normal colon, as well as HBEpiC, isolated from normal bronchial tissue. An anti-CD3 monovalent bispecific antibody (BsAb) as well as a human immunoglobulin G1 (hIgG1) isotype control (CTRL) were used as comparators. Ab, antibody; nM, nanomolar; CTRL, control.


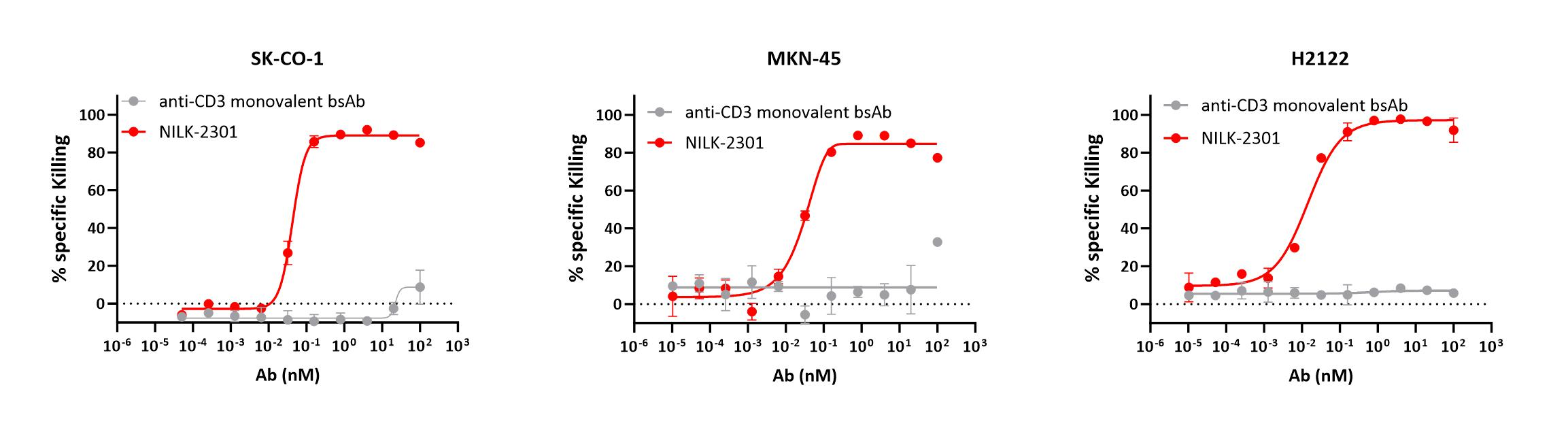


**Supplementary Figure S4. TDCC assay with CEACAM5-positive cell lines based on ATP quantification after 72 hours of co-culture.** Longer incubation with NILK-2301 leads to higher levels of specific lysis close to 100%. BsAb, bispecific antibody; Ab, antibody; nM, nanomolar.


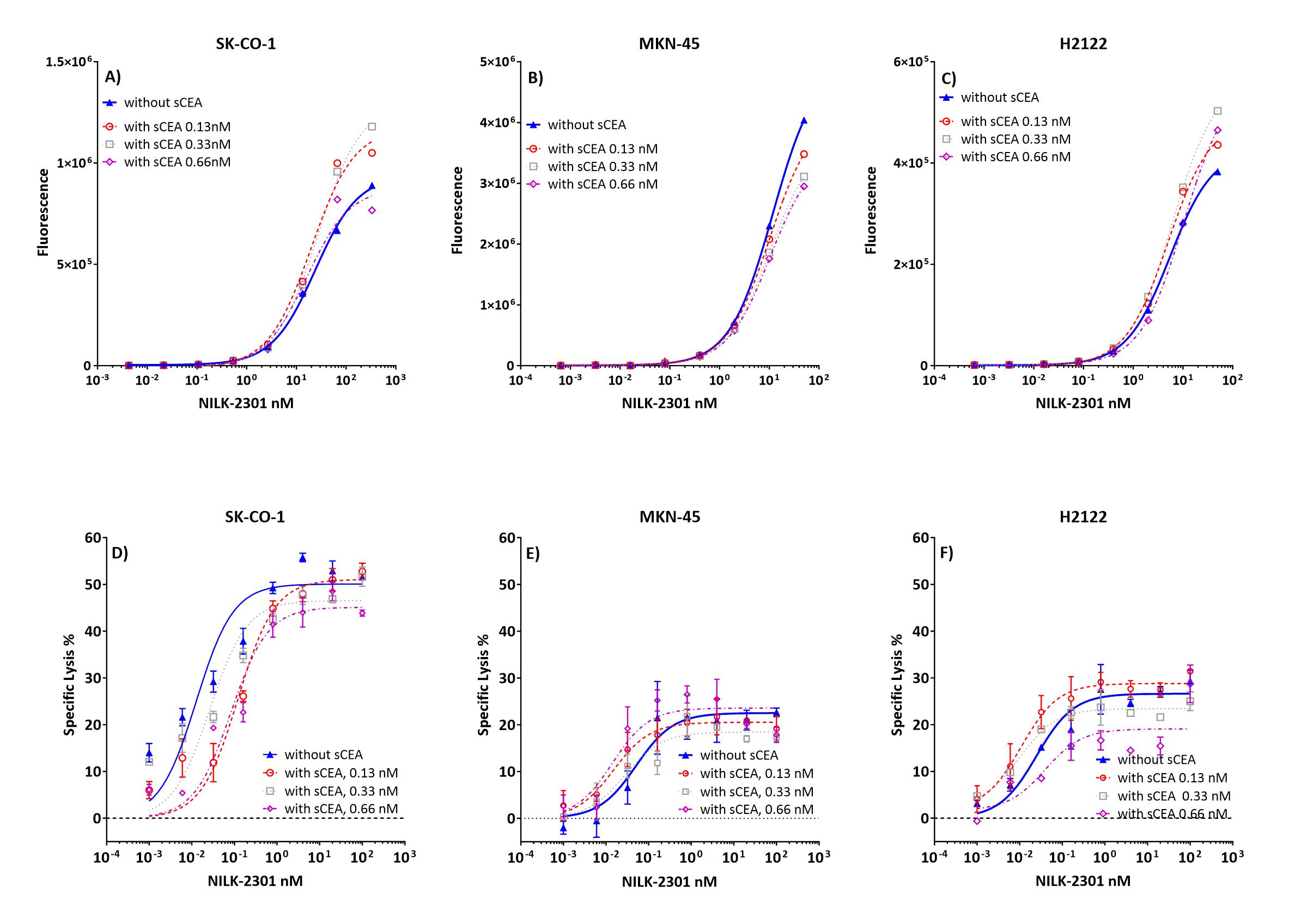


**Supplementary Figure S5. Impact of sCEACAM5.** Binding of NILK-2301 to A) SK-CO-1, B) MKN-45, and C) H2122, as well as D-F) corresponding TDCC activity. sCEA, soluble CEACAM5; nM, nanomolar.


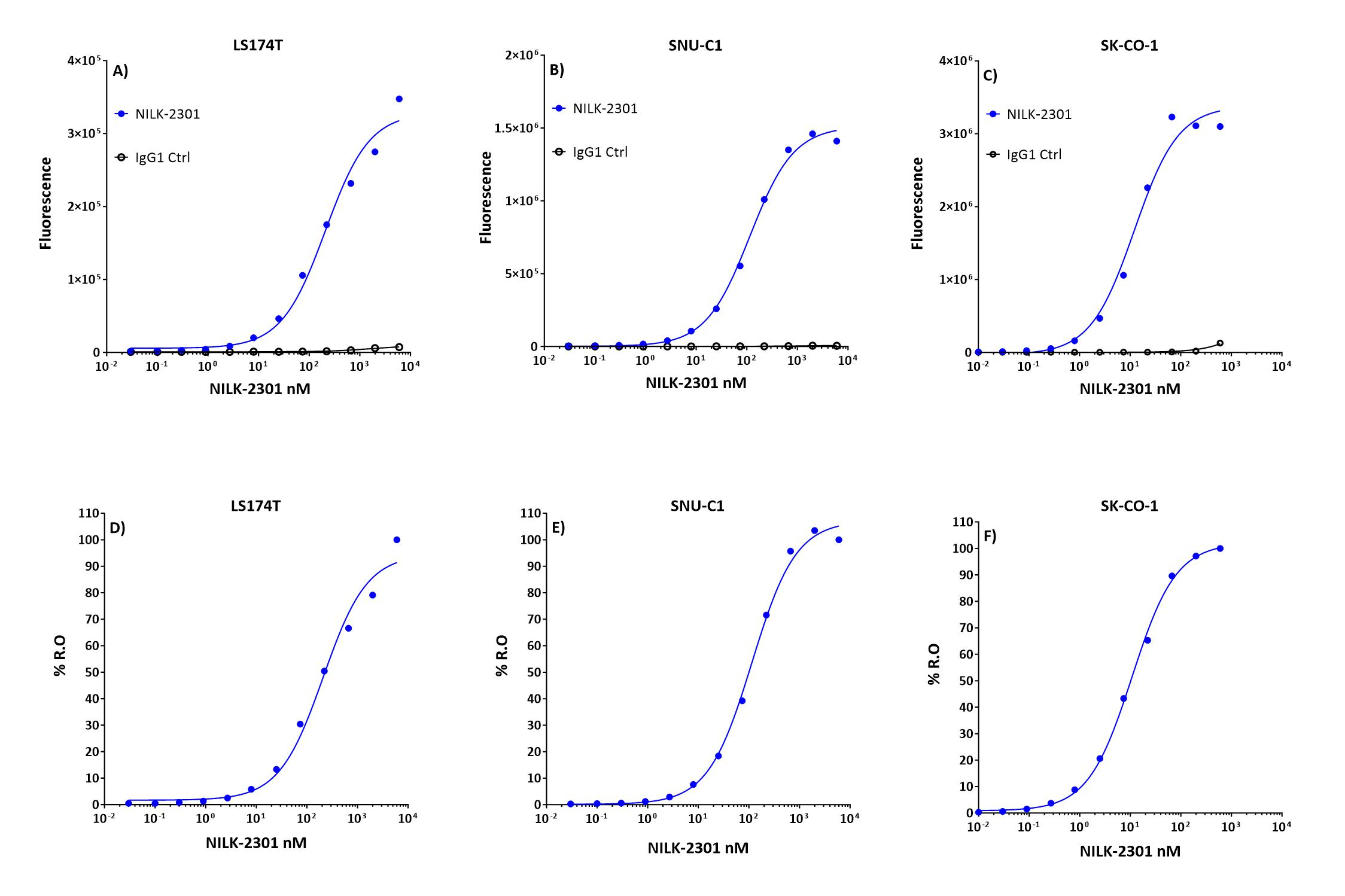


**Supplementary Figure S6. Binding of NILK-2301 to CEACAM5-positive cell lines and corresponding receptor occupancy.** Binding was assessed for colorectal cancer cell lines with different CEACAM5-expression levels, i.e., A) LS174T, B) SNU-C1, and C) SK-CO-1 by using flow cytometry. Corresponding percentage of receptor occupancy (% R.O.) of NILK-2301 was measured for D-F) LS174T, SNU-C1, and SK-CO-1. An inverse correlation between the level of CEACAM5 expression on the target cells and the RO_50_ of NILK-2301 to the target cells was observed. nM, nanomolar.

Supplementary TABLES

**Supplementary Table S1. Reference of commercial antibodies used as positive control to verify expression on PEAK cells.** The expression level of each CEACAM on PEAK cells was assessed before the binding experiment by using flow cytometry. See also Supplementary Figure S2.

**Supplementary Table S2. Quantification of CEACAM5-surface expression.** Cell lines and normal epithelial cells used with their tissue origin and number of surface CEACAM5 molecules/cell as assessed by QIFIKIT® assay.

*Isolated from non-cancerous human tissue.

**Supplementary Table S3. Mean background corrected Caspase3/7 fluorescence intensity values.** Table summarizing the mean background corrected Caspase3/7 fluorescence intensity values with their standard deviations (SD) overtime. Co-cultures were treated with 0.3, 1, and 5 µg/mL of NILK-2301 or a monovalent anti-CD3 antibody, respectively. Staurosporine at 10 µM was used as positive control.

**Supplementary Table S4. Summary of the mean value of the normalized tumor response in PDTC specimens.** Column 2 gives the CEACAM5 expression level of the tumor cells on a three level scale, with absence of CEACAM5 expression indicated by 0 and high CEACAM5 expression by 2. Red color indicates samples with response of ≥15%. Blue color indicates samples which showed responses of ≥10-14%.

Pembro, Pembrolizumab (anti-PD1 antibody); Nivo, Nivolumab (anti-PD1 antibody); Ipi, Ipilimumab (anti-CTLA-4 antibody); NA, not available.

**Supplementary Table S5. PK parameters for NILK-2301 after IV administration.** Data are shown for A) 0.5 mg/kg and B) 10 mg/kg in cynomolgus monkeys.

* One condition on PK parameters was not fulfilled.

**Supplementary Table S6. PK parameters for NILK-2301 after SC administration at 20 mg/kg** in cynomolgus monkeys.

* At least one condition on PK parameters was not fulfilled.

^§^ Animals were re-used after IV dosing.
